# Calcium overload induced mitochondrial and lysosomal dysfunction is regulated by Tousled-like kinase in a-synucleinopathy

**DOI:** 10.1038/s41419-025-08213-8

**Published:** 2026-01-08

**Authors:** Fangyan Gong, Qi Cheng

**Affiliations:** 1 Department of Neurology, Hubei No.3 People’s Hospital of Jianghan University, Wuhan, 430060 China; 2 Clinical Research Center, The Affiliated Wuxi People’s Hospital of Nanjing Medical University, Wuxi, 214023 China

**Keywords:** Cell death in the nervous system, Parkinson's disease

## Abstract

As a pathological hallmark of Parkinson’s disease (PD), a-synucleinopathy induces various cellular damages, including calcium overload, mitochondrial and autophagic dysfunction, ultimately resulting in dopaminergic neuron death. However, the hierarchy of these detrimental events remains unclear. It is well established that a-synuclein can induce calcium overload through diverse mechanisms. To assess whether calcium overload plays a crucial detrimental role, we established a calcium overload model in *Drosophila* and conducted genetic screening. Our findings indicate that calcium overload caused mitochondrial damage and lysosomal dysfunction, leading to cell death, and these cytotoxic processes were significantly mitigated by the loss of Tousled-like kinase (TLK). Notably, the loss of TLK also ameliorated defects induced by a-synuclein overexpression in *Drosophila*. This suggests that calcium overload is a critical event in a-synucleinopathy. In mammalian cells and mice, calcium overload activated TLK2 (the homologue of *Drosophila* TLK) by enhancing TLK2 phosphorylation, which increases TLK2 kinase activity. Increased TLK2 phosphorylation was detected in the brains of GluR1^Lc^ and a-synuclein overexpression mice, suggesting that TLK2 is activated under these pathological conditions. Furthermore, TLK2 knockout mice exhibited rescue of multi-aspect cytotoxicity induced by calcium overload and a-synuclein overexpression. Our research demonstrates that TLK2 activation by calcium overload appears to be a pivotal step in the progression of PD. This finding provides a potential link between calcium overload, the subsequent mitochondrial and lysosomal dysfunction observed in the disease.

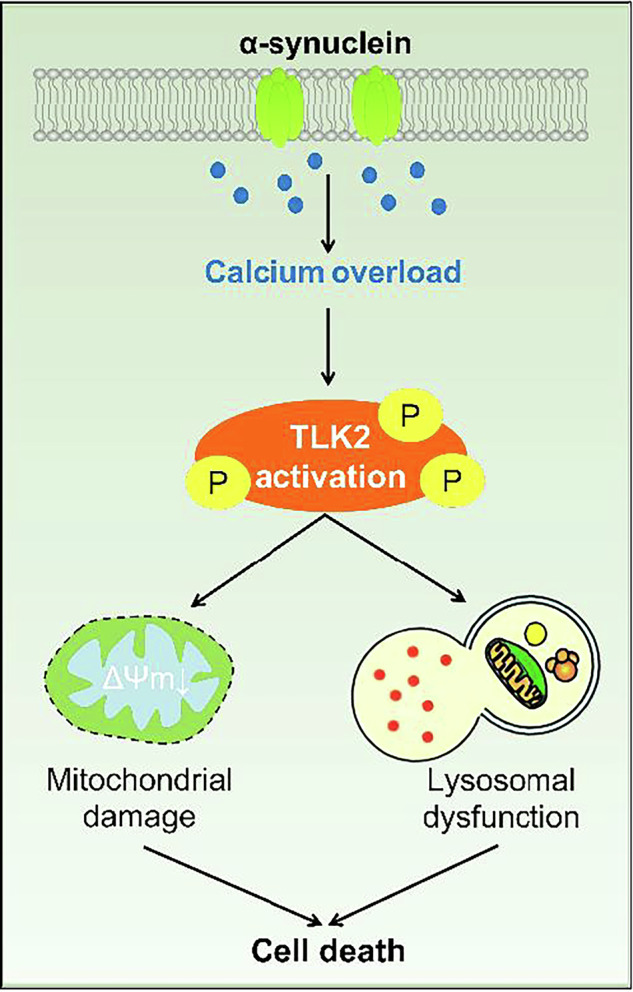

## Highlights

- Calcium overload induces mitochondrial damage, lysosomal dysfunction, and cell death in a-synucleinopathy.
- Loss of Tousled-like kinase (TLK) mitigates calcium-overload-induced and a-synuclein-induced cytotoxicity.
- Calcium overload activates TLK2 by enhancing its phosphorylation and kinase activity.
- TLK2 activation by calcium overload appears to be a pivotal step in Parkinson’s disease progression.

## Introduction

Parkinson’s disease (PD) is a common neurodegenerative disorder affecting 2-3% of individuals aged over 65 years old. The prominent pathological hallmark in the brain of PD patients comprise in a-synuclein in dopamine (DA) neurons [1]. a-synuclein causes multi-aspect of pathological damages, including aberrant protein aggregates, disruption of autophagy-lysosome catabolism, mitochondrial damage and disruption of calcium homeostasis [2]. Recent studies have provided compelling evidence suggesting that the loss of calcium homeostasis plays a prominent pathological role in PD [3]. For instance, pacemaking activity in DA neurons leads to sustained Ca^2+^ influx through CaV1.3, an L-type voltage-dependent Ca^2+^ channel, which renders neurons vulnerable to stress [4]. Blocking CaV1.3 by isradipine, a dihydropyridine blocker approved for hypertension treatment, had a protective effect in animal models of PD, although it failed in a clinical trial [5]. In fact, intracellular Ca^2+^ elevation may also be initiated by a variety of cation channels on the cytoplasmic, ER or mitochondrial membranes. Moreover, both extracellular and intracellular a-synuclein oligomers can form Ca^2+^-permeable pores to elevate intracellular Ca^2+^ concentration and cell death [6, 7]. In addition, a-synuclein fibers may alter the packaging of lipid membranes to disrupt cytosolic calcium homeostasis [8]. Alternatively, a-synuclein oligomers may provoke inositol 1,4,5-triphosphate receptor (IP3R) in the ER, impair mitochondrial respiratory complex I function and lysosomes, contributing to cytosolic Ca^2+^ overload [3, 9]. Compelling evidence suggests that calcium overload plays a key detrimental role in inducing and propagating the pathogenesis of PD. Cathepsin D and calcineurin are known regulators downstream of calcium overload under a-synuclein expression, and partial inhibition of calcineurin is beneficial for neuronal survival [10, 11]. The existence of other key regulators downstream of calcium overload remains unknown.

In addition to calcium overload, most PD-related genes, such as a-synuclein, PINK1, Parkin, LRRK2, DJ-1, GBA and ATPA13A2, are involved in mitochondrial function and autophagy-lysosome pathways, and mutations in these genes cause diverse alterations related to the progression of PD [12, 13]. Drugs targeting mitochondrial function or the autophagy-lysosome pathway have shown a wide spectrum of protective effects in animal models of PD, however, they have all failed to slow the progression of PD in patients [12, 14].

Among the detrimental mechanisms of calcium overload, mitochondrial dysfunction and autophagy-lysosome deficiency, which one is the initial inducer? Is there a crucial regulator that initiates these damages? To address these questions, we generated a *Drosophila* model of calcium overload and performed genetic screening to identify genetic modifiers. Then, we tested the modifiers in an a-synuclein overexpression induced PD model in *Drosophila*. The results showed that calcium overload caused cell death associated with both mitochondrial and autophagy-lysosomal dysfunctions. Strikingly, loss-of-function of Tousled-like kinase (TLK) abolished both calcium-overload-induced and a-synuclein-induced cytotoxicity, suggesting that TLK is a key regulator of multi-aspect pathological events in PD. Using in vitro biochemical analysis and mouse genetics, we further determined that calcium overload induced hyper-phosphorylation of TLK2. We hypothesize that phosphorylated TLK2 may be a key factor in inducing mitochondrial damage and lysosomal dysfunction after calcium overload.

## Results

### Calcium overload in *Drosophila* induces diverse cytotoxicity and cell death

**Fig. 1:**
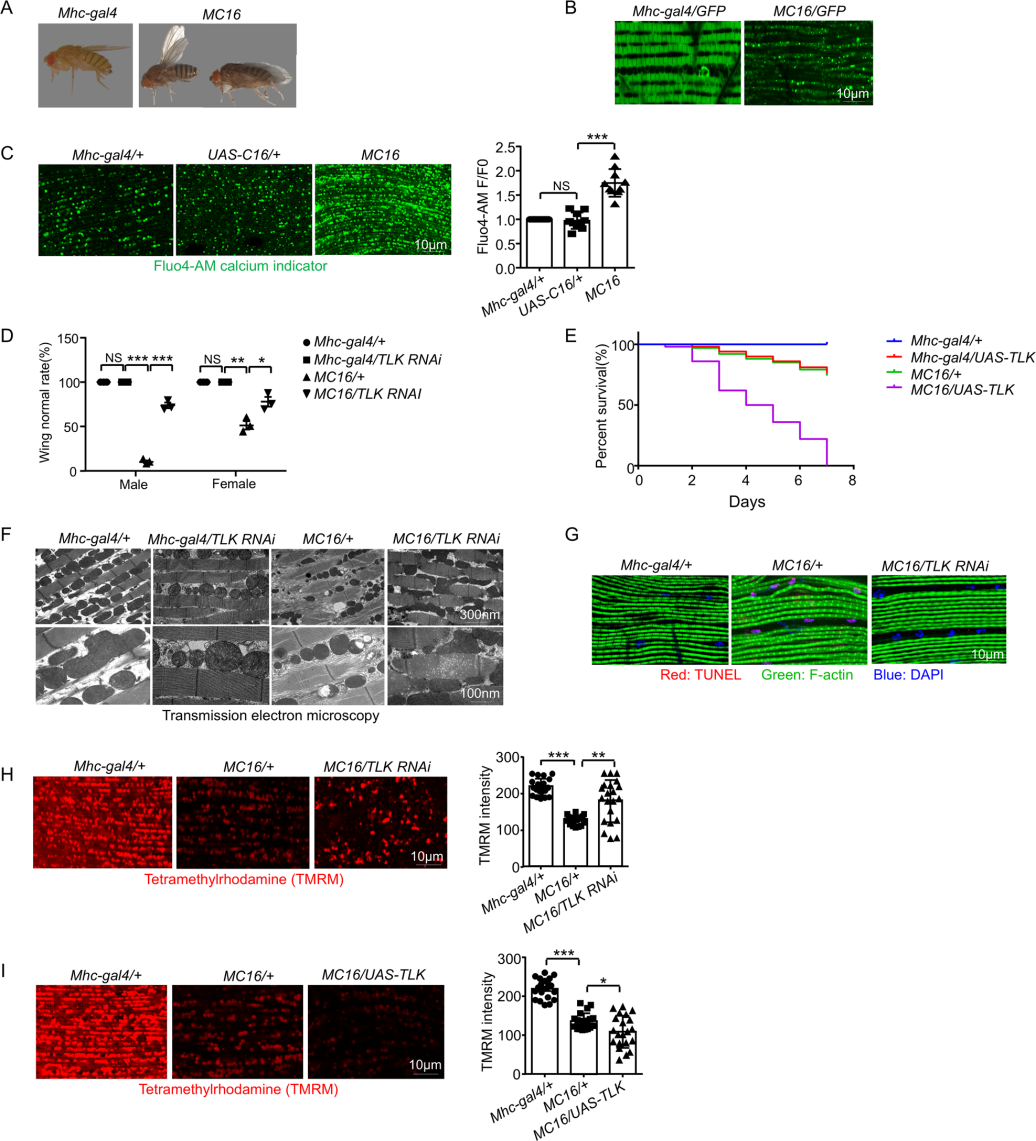
*MC16* is a calcium overload model in *Drosophila* and *TLK RNAi* reduces the diverse cytotoxicity of *MC16* fly. **A** The wing phenotype of *MC16* fly. Images are representative of three flies with the indicated genotypes. **B** Live imaging of the indirect flight muscle morphology of adult flies. The muscles were labeled with *UAS-GFP* driven by *Mhc-gal4*. Images are representative of three flies with the indicated genotypes. Scale bar, 10 μm. **C** Calcium overload level was measured by Fluo4-AM (5 μM, green) calcium indicator, the relative intensity was analyzed by F/F0, F represented the fluorescence intensity (*n* = 10 tissues from 10 flies). Scale bar, 10 μm. Error bars indicate SD. Statistical significance was performed with an unpaired *t*-test between two groups. For comparison of more than two groups, significance was determined using a one-way ANOVA with Tukey’s post hoc test. Kaplan–Meier tests were used under the log-rank algorithm for survival analysis. *P < 0.05, **P < 0.01, ***P < 0.001 and ****P < 0.0001. **D** The wing normal rate of the *Mhc-gal4/+, Mhc-gal4/TLK RNAi, MC16/+* and *MC16/TLK RNAi* flies. Both male and female flies were compared; 100 flies were tested for each trial (*n* = 3 independent trials). **E** The survival rate of 3-7 days of *Mhc-gal4/+, Mhc-gal4/UAS-TLK, MC16/+* and *MC16/UAS-TLK* flies were analyzed. 100 flies were tested for each trial (*n* = 3 independent trials). **F** Transmission electron microscopy (TEM) images of the indirect flight muscle of adult flies (*n* = 5 tissues from 5 flies). Scale bar, 300 nm and 100 nm. **G** TUNEL (red) staining of the indirect flight muscle, muscles were stained with F-actin (green). Nuclei were stained with DAPI (blue). Scale bar, 10 μm. Images are representative of six flies with the indicated genotypes. **H**, **I** Tetramethylrhodamine (TMRM, red) staining of the indirect flight muscle. The fluorescence intensity was analyzed (*n* = 20 tissues from 20 flies). Scale bar, 10 μm.

The cytotoxicity induced by a-synuclein shares many features with calcium overload induced damage, including mitochondrial dysfunction, autophagy-lysosome defect, ER stress and cell death [3, 15]. To test whether calcium overload plays a crucial detrimental role, we generated a transgene to express a mutant (G430C) of human brain sodium channel 1 (BNC1, also known as ASIC2a), which forms a constitutively opened non-selective cation channel in heterologous expression systems [16]. Therefore, the expression of BNC1_G430C_ is more likely to induce calcium toxicity over sodium toxicity. We generated a BNC1_G430C_ transgenic fly, simplified as *UAS-C16* [17]. For genetic screens, we tested different promoters and observed that *Mhc-gal4* (a muscle promoter) driven *C16* (*Mhc-gal4* > *C16*, or *MC16*) resulted in stereotypical wing posture defects in adult flies, a phenotype that is easy to quantify (Fig. 1A). When crossed with *UAS-GFP*, muscle damage was observed (Fig. 1B). Using the cell-permeable dyes Fluo4-AM and Fura2-AM, which are calcium indicators, we confirmed that the cytoplasmic Ca^2+^ level of *MC16* flies was increased compared to that of the control (Fig. 1C and Supplementary Fig. S1A). This indicates that *MC16* is a model of calcium overload. Meanwhile, the lifespan of *MC16* flies was shortened (Supplementary Fig. S1B).

### TLK is a key regulator of calcium overload in *Drosophila*

To screen for genetic modifiers against the calcium-overload-mediated cell damage, the wing posture defects were quantified. We observed that *MC16* male flies displayed a higher percentage of wing defects than female flies (Fig. 1D). We screened approximately 437 UAS-RNAi and candidate-based lines (Supplementary Table S1) and identified genetic modifiers of the following genes: Raptor, CalpB, MED7, MED19, MED20, MED27, TLK, Crtc, Atg8a and Reptor. The RNAi of Crtc, Atg8a and Reptor genes were enhancers, and the RNAi of Raptor, CalpB, MED7, MED19, MED20, MED27 and TLK were suppressors (Supplementary Table S2). Among them, we found Tousled-like kinase (TLK) RNAi showed the most striking rescue effect (Fig. 1D). Interestingly, overexpression of TLK could enhance *MC16* lethality (Fig. 1E), suggesting that TLK is necessary and sufficient to regulate calcium overload induced cell death. Because many RNAi can have off-targets, we used two TLK RNAi lines that targeted different TLK mRNA sequences. The effects of TLK RNAi on TLK transcription were confirmed by qRT-PCR, and they did not affect the expression of *C16* (Supplementary Fig. S1C, D). In addition, TLK RNAi could rescue the muscle morphology and mitochondrial defects in the *MC16* flies (Fig. 1F). We also determined cell death in *MC16* flies using TdT-mediated dUTP nick end labelling (TUNEL) and propidium iodide (PI), which detect apoptosis and necrosis respectively. Compared to the negative staining in the control flies, TUNEL signals were present in the muscle cells of *MC16* flies, and TLK RNAi strongly inhibited cell death (Fig. 1G and Supplementary Fig. S1E). PI staining was negative in *MC16* flies, indicating that the plasma membrane was intact (Supplementary Fig. S1F). Tetramethylrhodamin (TMRM) is a fluorescent dye that can be sequestered by active mitochondria, its positive staining indicates healthy mitochondria. Compared to the control flies, the fluorescence of TMRM was greatly reduced in the *MC16* flies, and this defect was restored by TLK RNAi (Fig. 1H) and was enhanced by the overexpression of TLK (Fig. 1I). TLK RNAi and TLK overexpression did not affect calcium overload (Supplementary Fig. S1G), suggesting that TLK is a key regulator of calcium overload.

### Cellular damage induced by α-synuclein overexpression is rescued by TLK RNAi in *Drosophila*

**Fig. 2:**
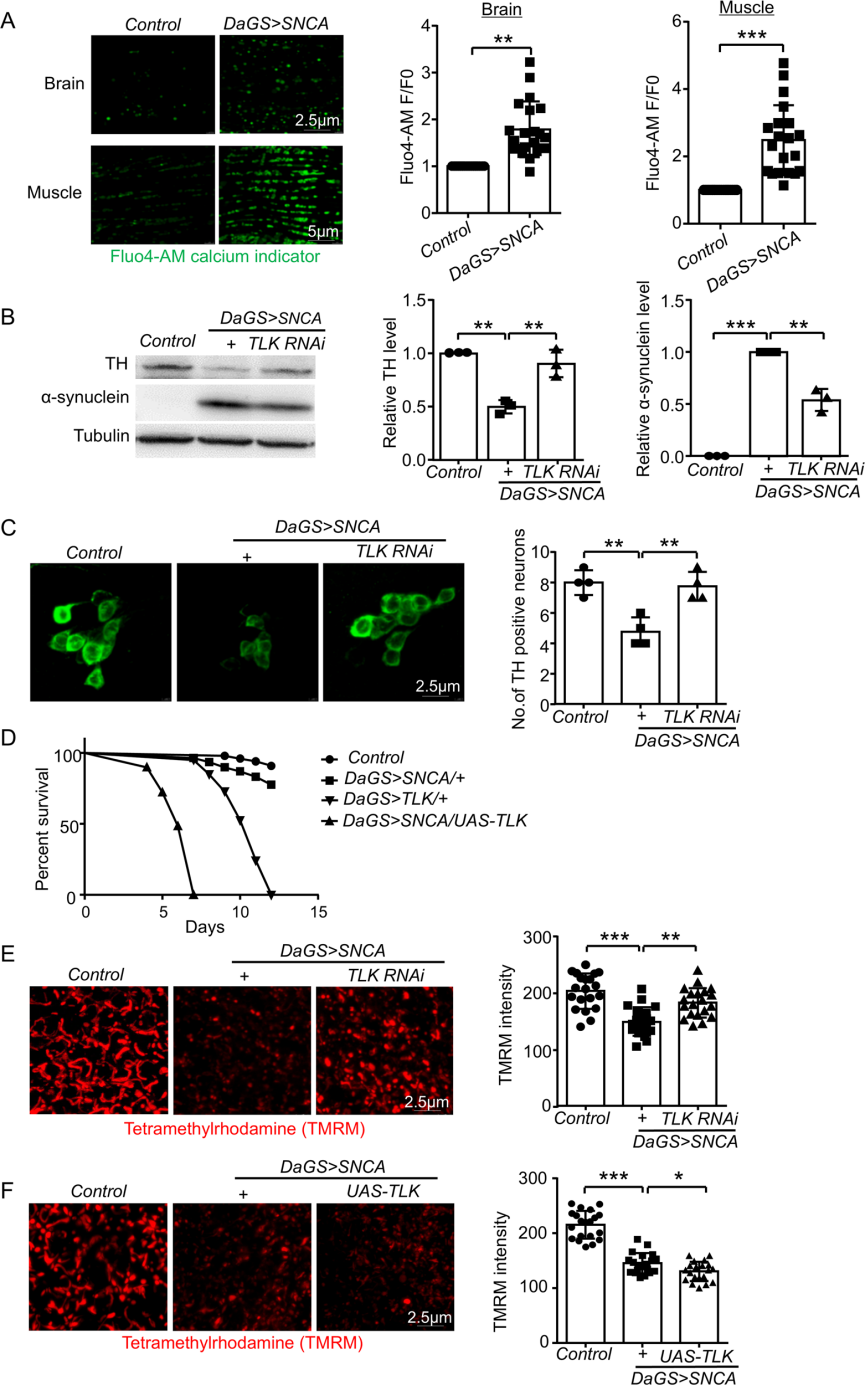
*TLK RNAi* rescues the α-synuclein overexpression induced cellular damage in *Drosophila.* **A** Calcium overload level was measured by Fluo4-AM (5 μM, green) calcium indicator of the brain and the muscle in 30 days-old, the relative intensity was analyzed by F/F0, F represented the fluorescence intensity (*n* = 20 tissues from 20 flies). Brain Scale bar, 2.5 μm, Muscle Scale bar, 5 μm. **B** Western blot of the tissue extracts from the whole body in 30 days-old. Quantification of TH and α-synuclein level. 20 flies were tested for each trial (*n* = 3 independent trials). **C** Immunostaining images of the TH neurons (green) in *DaGS* > *SNCA* fly brain PPL1 cluster. Scale bar, 2.5 μm. 5 flies were tested for each trial (*n* = 4 independent trials). Quantification of the number of the TH positive neurons. **D** The lifespan changes of *Control* (No RU486), *DaGS* > *SNCA/+*, *DaGS* > *TLK/+* and *DaGS* > *SNCA/UAS-TLK* flies. 80-100 flies were tested for each trial (*n* = 3 independent trials). **E**, **F** TMRM (1 uM, red) staining of the fly brain, the fluorescence intensity was analyzed (*n* = 20 tissues from 20 flies). Scale bar, 2.5 μm.

To test whether a-synuclein overexpression induces calcium overload, we obtained a *Drosophila* transgenic line that expresses a-synuclein. The *UAS-SNCA* (the a-synuclein gene) was driven by a gene switch inducible promoter *daughterless-gal4 (DaGS)* [18]. The progeny flies were simplified as *DaGS* > *SNCA*. Adult flies were fed with RU486 (mifepristone) to induce the Gal4 expression ubiquitously. Since RU486 did not affect fly health [19], *DaGS* > *SNCA* flies fed with RU486 were compared with the control (*DaGS* > *SNCA* flies without feeding RU486). In 20 days-old of *DaGS* > *SNCA* flies, a-synuclein could not induce calcium overload (Supplementary Fig. S2A), indicating a-synuclein might not reach the threshold level. In these flies, TLK RNAi could not reduce a-synuclein level and restore the Tyrosine Hydroxylase (TH) level, a marker of DA neuron (Supplementary Fig. S2B). In 30 days-old *DaGS* > *SNCA* flies, intracellular Ca*2+* level was increased in brain and muscle cells (Fig. 2A), indicating that a-synuclein promotes calcium overload. The expression of a-synuclein was detectable and associated with reduced TH level (Fig. 2B), suggesting that a-synuclein overexpression causes DA neuron death. Importantly, TLK RNAi reduced a-synuclein protein level and restored TH protein level in *DaGS* > *SNCA* flies (Fig. 2B). TH immunostaining also showed rescue effects (Fig. 2C and Supplementary Fig. S1C). These results indicate that TLK RNAi may be effective in cell death induced by a-synuclein expression. In addition, we found that TLK overexpression further shortened the lifespan of flies expressing a-synuclein (Fig. 2D). Furthermore, defective mitochondrial function in *DaGS* > *SNCA* flies was restored by TLK RNAi (Fig. 2E) and enhanced by TLK overexpression (Fig. 2F), and TLK RNAi did not affect calcium overload level induced by a-synuclein expression (Supplementary Fig. S2D), and mRNA level of SNCA (Supplementary Fig. S2E). These results suggest that a-synuclein overexpression may cause mitochondrial damage, leading to cell death through TLK activation, which is induced by calcium overload.

### Calcium overload disrupted lysosomal function via TLK

**Fig. 3:**
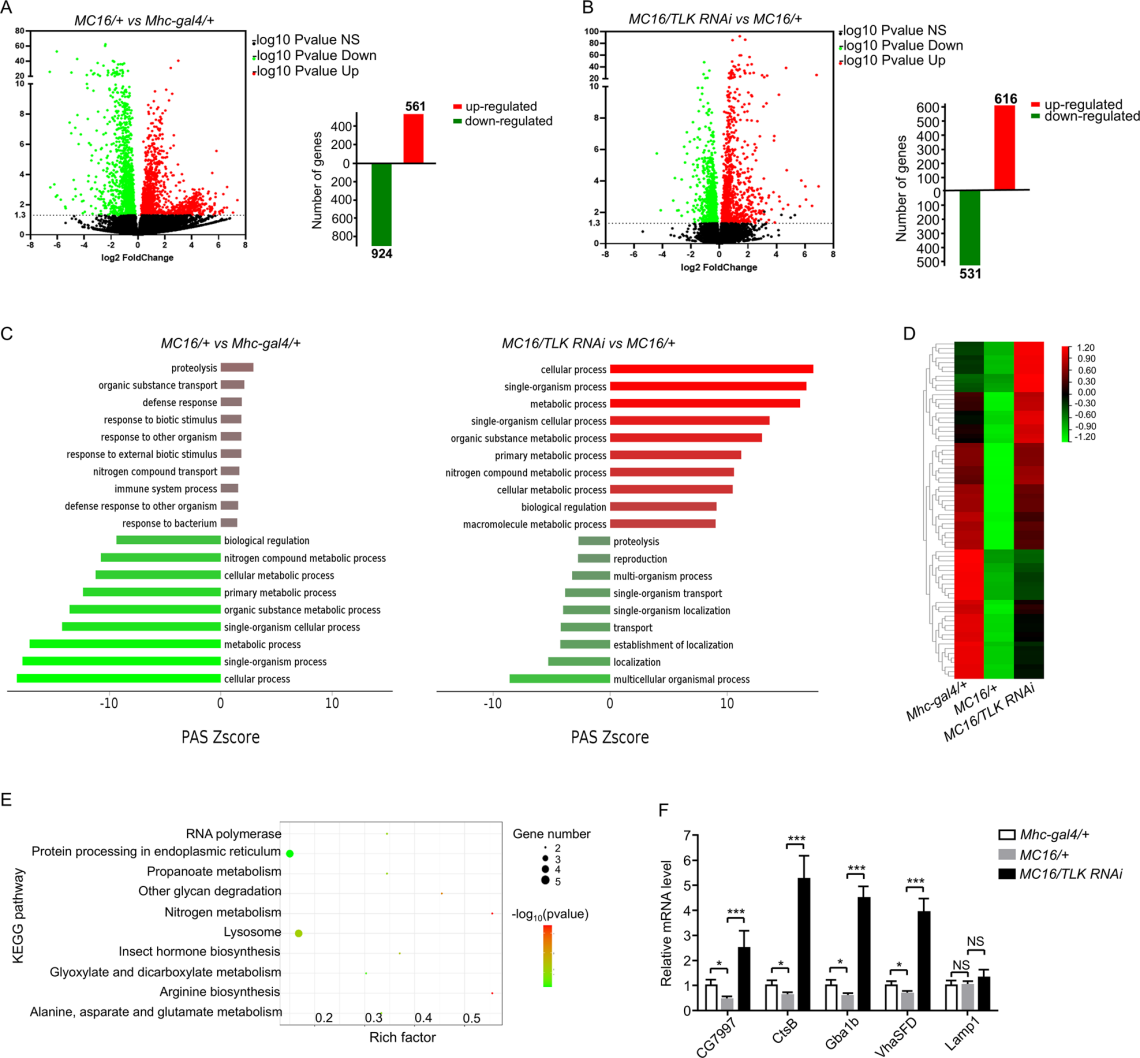
Analysis of global transcriptional changes in *Mhc-gal4/+*, *MC16/+* and *MC16/TLK RNAi* flies. **A**, **B** The volcano plot of gene expression difference between groups of flies as indicated on the graph with *p*-value < 0.05. The red dots indicate upregulated genes, green dots indicate the downregulated genes, 561 genes upregulated and 924 genes downregulated in the *MC16* flies. Compared the *MC16* flies, TLK RNAi under *MC16* background results in 616 genes upregulated and 531 genes downregulated. Three independent samples were collected for each phenotype. **C** The enriched biological processes were evaluated by the Gene Ontology (GO). Green indicating the downregulated process and red indicating the upregulated process. **D**, **E** The heat map of RNA sequencing result. The overlapping genes of the downregulated genes in *MC16* and the upregulated genes in *MC16/TLK RNAi* were analyzed by KEGG pathways. The enriched pathways are indicated on the graph. **F** Quantitative RT-PCR analysis of five lysosomal genes mRNA expression from *Mhc-gal4/+*, *MC16/+* and *MC16/TLK RNAi* flies. 20 flies were tested for each trial (*n* = 3 independent trials).

To study the mechanism of calcium overload, we performed whole genome RNA sequencing to compare transcriptional alterations of the control (*Mhc-gal4*) and calcium overload (*MC16*) flies. The volcano plot displayed the differences in gene expression between the two groups of flies (Fig. 3A). 561 genes were upregulated and 924 genes were downregulated in the *MC16* flies (Fig. 3A). Compared with MC16 flies, TLK RNAi under the *MC16* background resulted in 616 upregulated genes and 531 downregulated genes (Fig. 3B). The enriched biological processes were evaluated by the Gene Ontology (GO). The result showed that genes involved in cellular process, single organism process and metabolic process were enriched with calcium overload, which reversed by the TLK RNAi (Fig. 3C). Then we focused on the 924 downregulated genes in *MC16* and 616 upregulated genes in *MC16/TLK RNAi*, and we found 73 genes overlapped, and their expression level were plotted (Fig. 3D). To further characterize the 73 genes, Kyoto Encyclopedia of Genes and Genomes (KEGG) analysis revealed that the lysosome and protein processing pathways in the endoplasmic reticulum (ER) were enriched (Fig. 3E). Using the fluorescent marker *UAS-ER-RFP* (label ER), we found that ER morphology showed no significant change (Supplementary Fig. S2F). Therefore, we focused on the lysosomal pathways. We performed qRT-PCR to quantify the transcripts of the five lysosomal genes identified from the RNA-seq. We found that TLK RNAi affected several lysosomal functional genes, including lysosomal acidification (CG7997, CtsB and Gba1b) and hydrolyzation (VhaSFD) gene, with no change in the Lamp1 gene (Fig. 3F). These results indicate that TLK is more likely to affect lysosomal function than biogenesis.

**Fig. 4:**
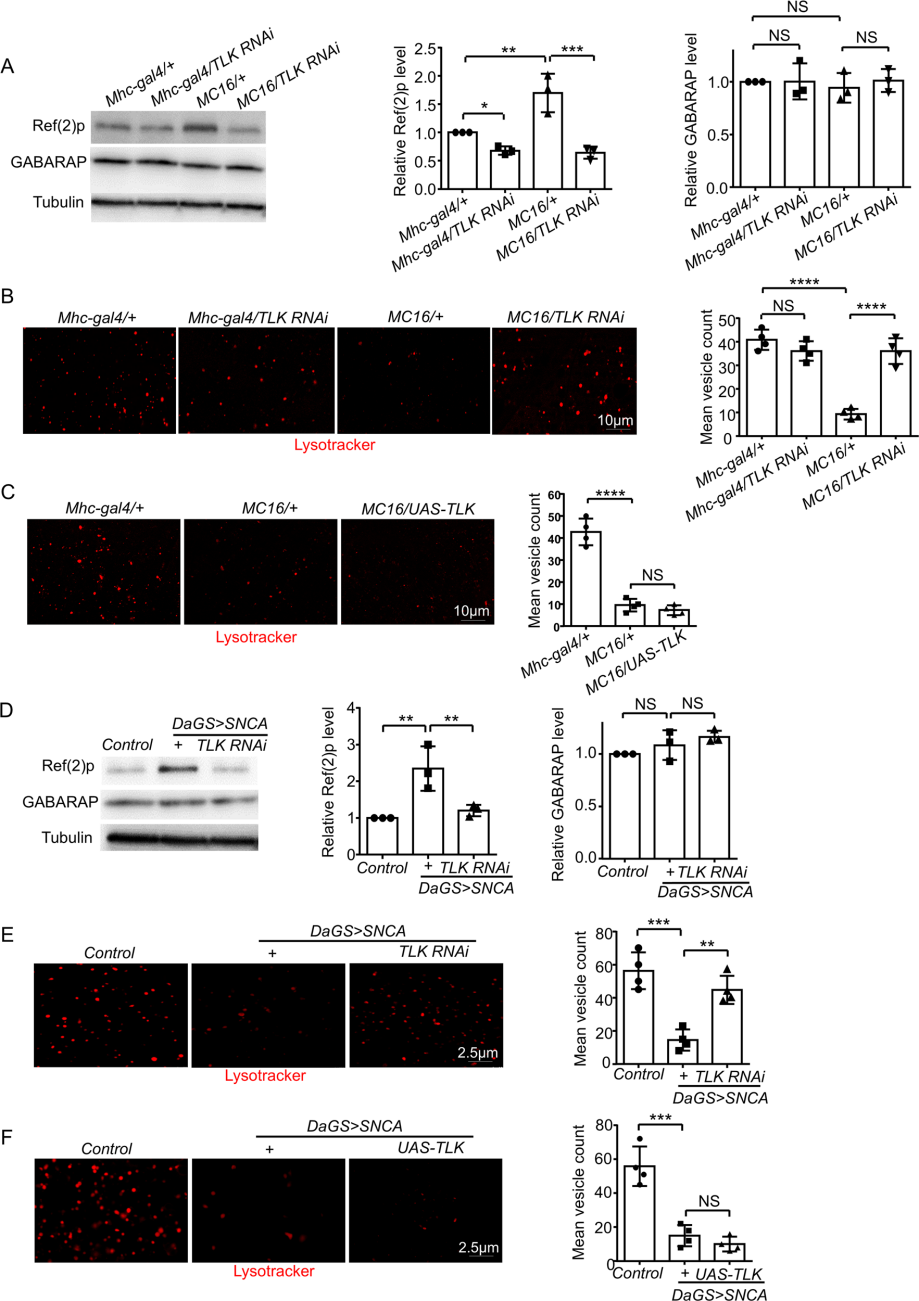
Calcium overload disrupted lysosomal pathway via TLK function. **A** Western blot of the tissue extracts from the indirect flight muscle. 20 flies were tested for each trial (*n* = 3 independent trials). Quantification of Ref(2)p and GABARAP level. **B**, **C** Lysotracker (red) staining of the indirect flight muscle. Scale bar, 10 μm. 5 flies were tested for each trial (*n* = 4 independent trials). Quantification of the number of lysotracker positive vesicles. **D** Western blot of the tissue extracts from the whole body. 20 flies were tested for each trial (*n* = 3 independent trials). Quantification of Ref(2)p and GABARAP level. **E**, **F** Lysotracker (red) staining of the fly brain. Scale bar, 2.5 μm. 5 flies were tested for each trial (*n* = 4 independent trials). Quantification of the number of lysotracker positive vesicles.

Next, we examined lysosome-autophagy status. As markers of autophagy, the LC3 protein complex is involved in the elongation of the phagophore membrane, and GABARAP protein is essential for autophagosome maturation [20]. In addition, Ref(2)P is the *Drosophila* homologue of mammalian P62, which functions as a substrate of autophagy to deliver ubiquitinated protein into autophagosome [21]. The accumulation of P62 indicates lysosomal dysfunction [22]. In *MC16* flies, Ref(2)P protein level was increased and GABARAP level was unaltered (Fig. 4A). This indicates that calcium overload mainly affects lysosomal function. Importantly, TLK RNAi rescued lysosomal dysfunction (Fig. 4A). Moreover, *MC16* flies showed decreased LysoTracker staining, which was rescued by TLK RNAi (Fig. 4B). TLK overexpression also enhanced this defect (Fig. 4C). This result further supports that lysosome activity is downregulated and this reduction depends on TLK function. Similarly, we observed that the Ref(2)P protein level was increased in RU486 fed *DaGS* > *SNCA* flies, which was associated with decreased LysoTracker staining (Fig. 4D, E), these deficits could also be rescued by TLK RNAi (Fig. 4D, E), and enhanced by TLK overexpression (Fig. 4F). This indicates that TLK RNAi can rescue lysosomal dysfunction downstream of calcium overload or a-synuclein overexpression in *Drosophila*. Collectively, the fly results demonstrated that calcium overload induced mitochondrial and lysosomal dysfunction is regulated by TLK in a-synucleinopathy.

### TLK2 KO rescued the mitochondrial damage and lysosomal dysfunction induced by calcium overload or α-synuclein overexpression

Vertebrate genomes encode two *Drosophila* TLK homologues, TLK1 and TLK2 [23]. Using the CRISPR/Cas9 system, we knocked out the human TLK1 and TLK2 genes in HeLa cells. To determine their effect on autophagy, we quantified the transcriptional changes in five lysosomal genes (GLA, CTSB, ATP6V1H, GBA and LAMP1) using qRT-PCR. The results showed that TLK2 KO, but not TLK1 KO, increased the transcription of three genes (GLA, CTSB and ATP6V1H) (Supplementary Fig. S3A, B). After induction of autophagy in Earle’s starvation buffer (EBSS) [24], these transcripts were further increased (Supplementary Fig. S3A). This indicates that TLK2, but not TLK1, regulates the lysosomal function. Therefore, we focused on TLK2 function. We compared the TLK protein sequence in *Drosophila* and TLK2 in mammals using the UniProt Knowledgebase (UniProtKB; https://www.uniprot.org/) (Supplementary Fig. S4). The results showed that the kinase domains of TLK and TLK2 were highly similar.

**Fig. 5:**
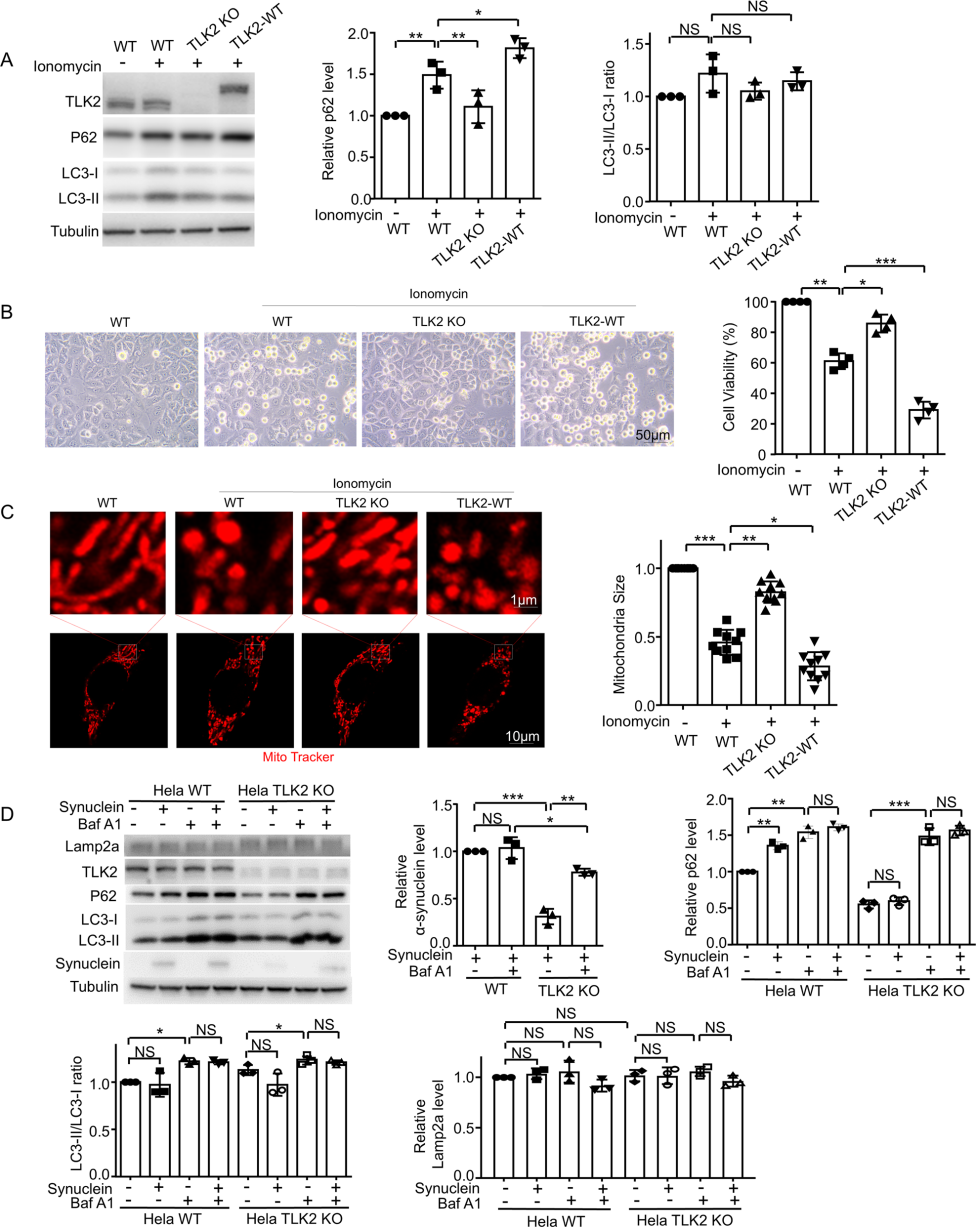
TLK2 KO rescued the mitochondrial damage and lysosomal dysfunction induced by calcium overload or α-synuclein overexpression. **A** Western blot of the extracts from Hela WT, TLK2 KO or TLK2 overexpression cells with or without treatment with ionomycin (1 μM for 24 h), Quantification of P62 and LC3-II/LC3-I ratio level (*n* = 3 independent trials). **B** Cell viability of Hela WT, TLK2 KO or TLK2 overexpression cells with or without treatment with ionomycin (1 uM for 24 h) (*n* = 4 independent trials). Scale bar, 50 μm. **C** Representative mitochondrial morphology stained with mito-tracker (red) of Hela WT, TLK2 KO or TLK2 overexpression cells (*n* = 10 independent trials). Scale bar, 10 μm and 1 μm. **D** Western blot of the extracts from Hela WT and TLK2 KO cells with Bafilomycin A1 (BafA1, 100 nM for 6 h) treatment. Using lentiviral transfection of α-synuclein, we established stable cell lines expressing α-synuclein in wild type Hela cells and TLK2 KO cells (*n* = 3 independent trials). Quantification of α-synuclein, P62, LC3-II/LC3-I ratio and Lamp2a level.

We knocked out or overexpressed TLK2 in HeLa cells using ionomycin to induce calcium overload. P62 level was increased in calcium overload cells compared to the WT cells, TLK2 KO could restore and TLK2 overexpression enhance the P62 level (Fig. 5A). LC3 II/I level showed no significant difference (Fig. 5A). This further proved that lysosomal dysfunction induced by calcium overload is regulated by TLK2. When treated with ionomycin to induce calcium overload, Hela WT cells died, TLK2 KO rescued the cell death, and TLK2 overexpression increased the cell death (Fig. 5B). Many studies have shown that calcium overload results in mitochondrial fission into small dots, indicating mitochondrial damage [25]. We also observed mitochondrial fragments in the calcium overload cells, which were rescued by TLK2 KO and enhanced by TLK2 overexpression (Fig. 5C). These data further showed that mitochondrial damage and lysosomal dysfunction induced by calcium overload are regulated by TLK2.

To study a cellular model of PD, we established stable cell lines expressing a-synuclein in wild-type Hela cells and TLK2 KO cells by lentiviral transfection of a-synuclein. Then we treated the cell lines with Bafilomycin A1 which inhibited the lysosomal function. The results showed that a-synuclein level decreased significantly under the TLK2 KO background compared with the wild type. Baf A1 treatment restored the a-synuclein level (Fig. 5D). We also detected the LC3 and P62 levels. We found that a-synuclein overexpression increased P62 levels in Hela WT cells but not in TLK2 KO cells (Fig. 5D), consistent with *Drosophila* data. In neuroblastoma SK-N-SH cells, a-synuclein overexpression showed a lower LC3-II level and higher P62 level compared to normal cells (Supplementary Fig. S3C), suggesting that a-synuclein overexpression reduces lysosomal function. This effect is consistent with that reported by others [26]. Interestingly, the downregulated lysosomal function could be rescued by a known TLK2 kinase inhibitor, promazine hydrochloride (PMZ) (Supplementary Fig. S3C). In addition, LysoTracker signal was decreased in a-synuclein overexpressed SK-N-SH cells, and PMZ rescued the lysosomal dysfunction (Supplementary Fig. S3D). These results indicate that TLK2 activation is involved in mitochondrial damage and lysosomal dysfunction induced by calcium overload or a-synuclein overexpression.

### Knockout TLK2 activates lysosome-autophagy function

**Fig. 6:**
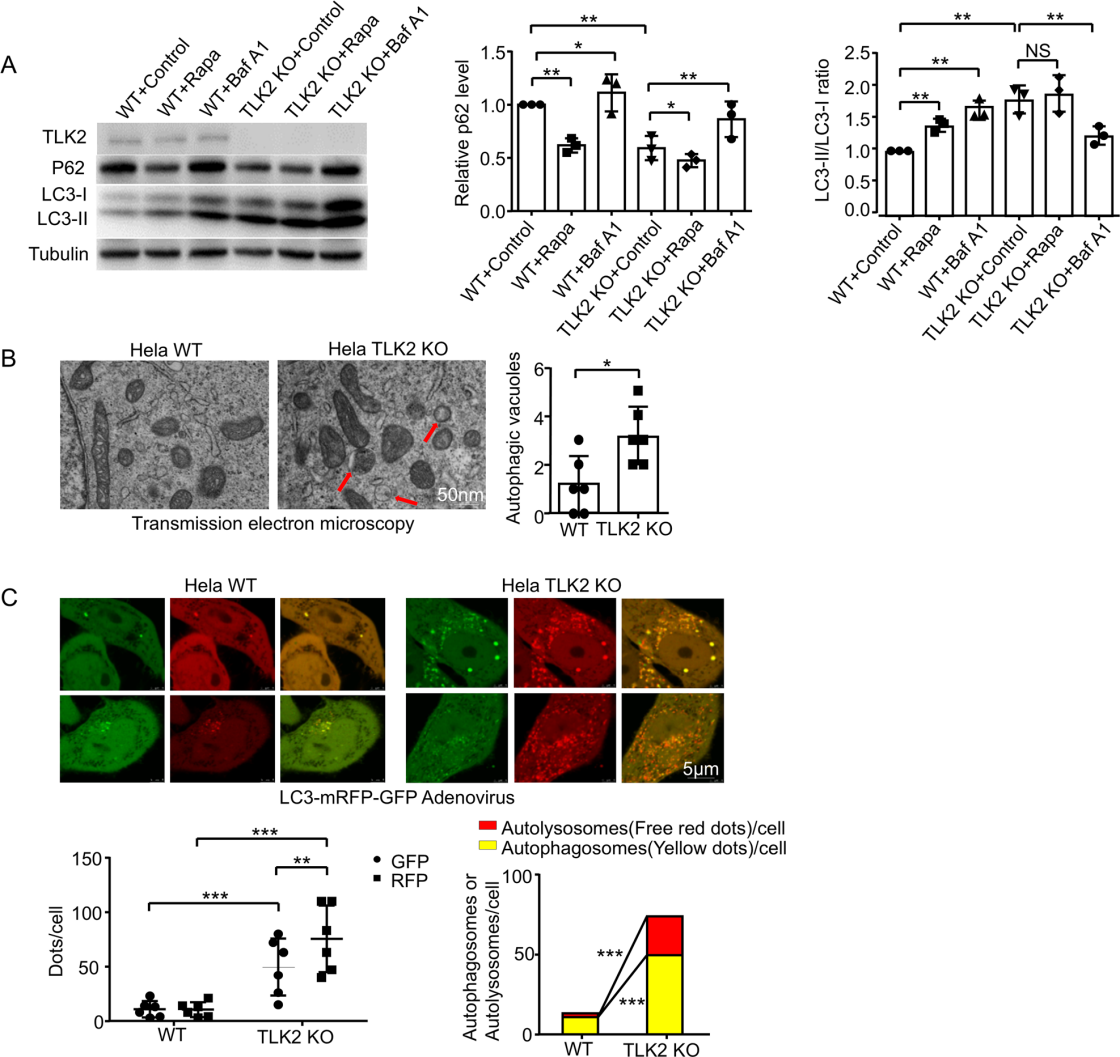
Knockout TLK2 activated lysosome-autophagy function. **A** Western blot of the extracts from Hela WT and TLK2 KO cells with Rapamycin (Rapa; 40 nM for 6 h) and Bafilomycin A1 (BafA1, 100 nM for 6 h) treatment (*n* = 3 independent trials). Quantification of P62 and LC3-II/LC3-I ratio level. **B** TEM images of the Hela WT and TLK2 KO cells (*n* = 6 independent trials). Scale bar, 50 nm. Quantification of the number of autophagic vacuoles (red arrows). **C** Live imaging of the Hela WT and TLK2 KO cells after LC3-mRFP-GFP adenovirus transfection 48 h (*n* = 6 independent trials). Scale bar, 5 μm. Quantification of the GFP and RFP dots, as well as autolysosomes (free red dots) and autophagosomes (yellow dots) per cell.

In TLK2 KO cells, the ratio of LC3-II/LC3-1was increased, associated with a decreased P62 level, but not in TLK1 KO cells (Fig. 6A and Supplementary Fig. S3E), suggesting that TLK2 KO cells activated autophagy. As a dynamic process, autophagic flux can be assessed by drug treatment to stop the flux at a certain stage. Bafilomycin A1 (Baf A1) inhibits the fusion of autophagosomes to lysosomes, rapamycin (Rapa) activates autophagy [24]. In wild-type Hela cells, Rapa treatment increased the LC3-II/LC3-1 ratio and decreased the P62 level, whereas Baf A1 treatment increased the LC3-II/LC3-1 ratio and P62 level (Fig. 6A). In the TLK2 KO cells, Rapa treatment further activated autophagy by decreasing P62 level, and the activated autophagy was abolished by Baf A1 treatment (Fig. 6A). Furthermore, transmission electron microscopy (TEM) imaging showed that more autophagosomes and autolysosomes were present in TLK2 KO cells than in wild-type cells (Fig. 6B). Another commonly used assay to measure autophagic flux is LC3-mRFP-GFP adenovirus infection [24]. The low pH inside the lysosome quenches the GFP fluorescent signal of LC3-mRFP-GFP, in contrast, RFP exhibits more stable fluorescence in acidic compartments. If autophagic flux is increased, both yellow and red puncta will increase. The yellow stains indicate autophagosomes and the red labels autolysosome. Indeed, both yellow and red puncta were increased in the TLK2 KO cells (Fig. 6C), suggesting increased autophagic flux. As additional support, P62 protein level was lower in P62 overexpression of TLK2 KO cells (Supplementary Fig. S3F). These results are consistent with a previous study of a genome-wide siRNA screen, in which siRNA TLK2 enhanced the amino acid starvation-induced autophagy [27]. Together, these results demonstrate that TLK2 KO activates lysosome-autophagy function.

### Calcium overload activates TLK2 by phosphorylation

**Fig. 7:**
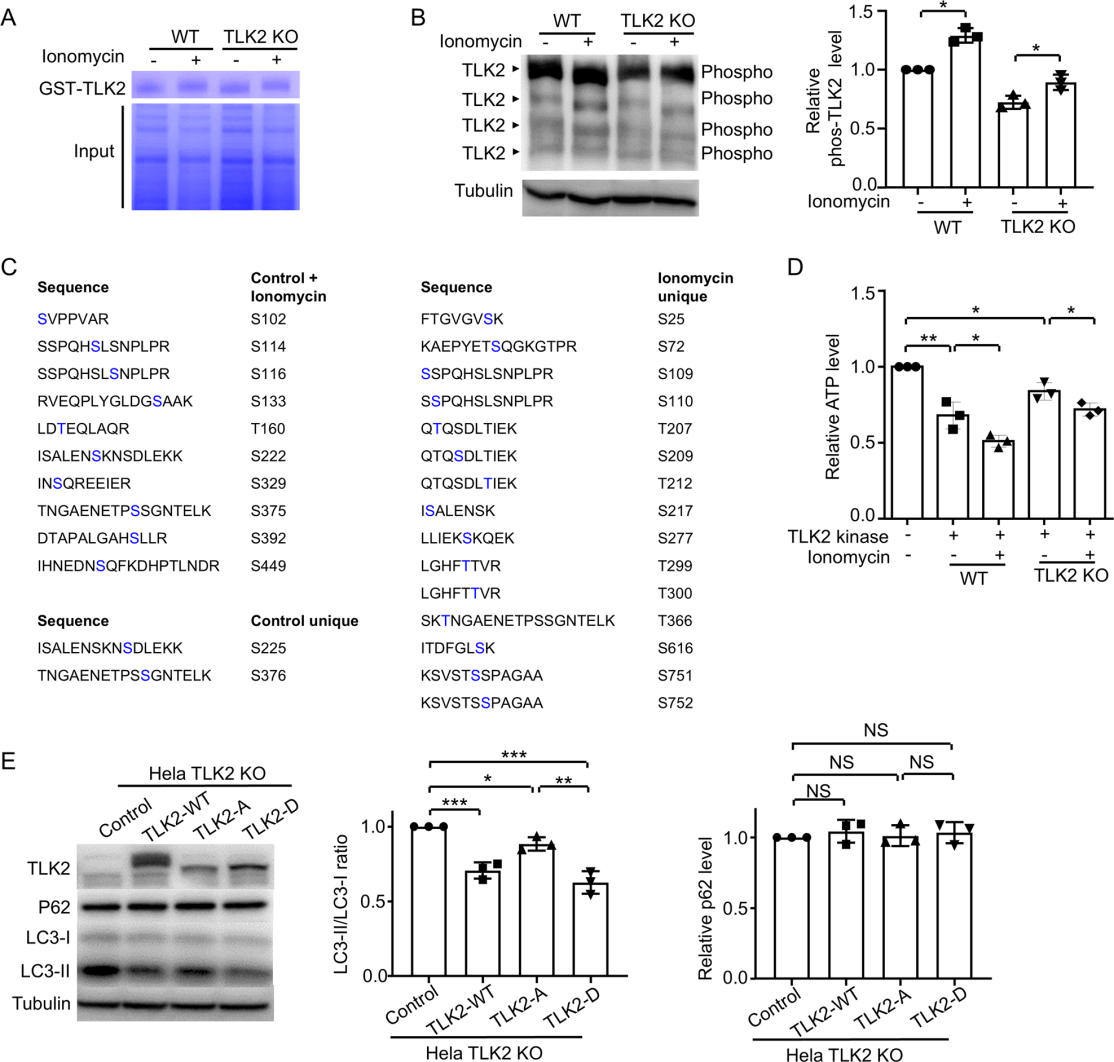
Calcium overload activated TLK2 by phosphorylation. **A** Coomassie Blue staining of the GST-TLK2 pull-down from the Hela WT and TLK2 KO cells with or without ionomycin (1 μM) treatment. Images are representative of three independent trials. **B** Phos-tag western blot of the phosphorylated GST-TLK2. Hela WT and TLK2 KO cells with or without ionomycin (1 μM) treatment. GST-TLK2 purification protein were incubated with the cell lysate and pulled down by Glutathione Sepharose 4B beads. Tubulin was used as a loading control (*n* = 3 independent trials). Quantification of TLK2 phosphorylation level. **C** Mass spectrometry results showed the phosphorylation sites after the incubation. Images are representative of three independent trials. **D** In vitro kinase assay, recombinant TLK2 protein in *E. coli* were purified. Myelin basic protein (MBP) was used as a substrate. The TLK2 kinase activity with or without ionomycin treatment were assessed by the reduced ATP level (*n* = 3 independent trials). **E** Western blot of the Hela TLK2 KO cell extracts after 48 h transfection of TLK2-WT (normal), TLK2-A (dephosphorylation), TLK2-D (phosphorylation) and control (*n* = 3 independent trials). Quantification of P62 and LC3-II/LC3-I ratio level.

TLK2 contains multiple phosphorylation sites and autophosphorylation can activate this enzyme [28]. To test whether TLK2 was phosphorylated during calcium overload, we constructed GST-TLK2 and pulled down TLK2 protein by GST. We purified TLK2 and incubated it with cell lysates from ionomycin-treated cells (induced calcium overload) or normal cells. Strikingly, we observed a shift of GST-TLK2 after incubation with ionomycin-treated cell lysate on the Coomassie Blue gel (Fig. 7A), indicating TLK2 protein is modified. In addition, incubation with ionomycin-treated cell lysate from TLK2 KO cells showed a similar shift, suggesting that the modification of TLK2 is not by itself (Fig. 7A). By phos-tag SDS-PAGE, we found TLK2 phosphorylation was increased (Fig. 7B). All serine/threonine phosphorylation sites of TLK2 were determined using mass spectrometry. In normal conditions (without ionomycin treatment), 12 sites were phosphorylated, including 10 sites shared in control and ionomycin-treated cells (S102, S114, S116, S133, T160, S222, S329, S375, S392 and S449), and 2 unique sites in control (S225 and S376). Treatment with calcium overload lysate resulted in 15 more phosphorylation sites (S25, S72, S109, S110, T207, S209, T212, S217, S277, T299, T300, T366, S616, S751 and S752), associated with the loss of 2 phosphorylation sites (S225 and S376) (Fig. 7C).

For the in vitro kinase assay, recombinant TLK2 protein in *E. coli* was purified. Myelin basic protein (MBP) has used as a substrate for many kinases [29]. In this kinase assay, calcium-overload-lysate-treated TLK2 resulted in increased activity in both WT and TLK2 KO lysates, as determined by ATP consumption (Fig. 7D). However, the TLK2 KO lysates showed less ATP decline than the WT lysates (Fig. 7D), indicating that TLK2 may phosphorylate itself. This result is consistent with a previous report suggesting that phosphorylation of TLK2 enhances its enzymatic activity [28]. Moreover, other unknown kinase(s) was activated by calcium overload and involved in phosphorylation of TLK2. Mass spectrometry revealed that TLK2 was phosphorylated at 15 sites, including S25, S72, S109, S110, T207, S209, T212, S217, S277, T299, T300, T366, S616, S751 and S752. We mutated these 15 sites to either alanine (A) to mimic dephosphorylation or aspartic acid (D) to mimic phosphorylation and expressed them in both wild-type and TLK2 KO HeLa cells. The results showed that overexpression of TLK2-WT or TLK2-D decreased the LC3-II/LC3-I ratio, but not overexpression of TLK2-A (Fig. 7E), indicating that the phosphorylated form of TLK2 suppresses autophagy-lysosome function. TLK2-WT migrated differently from TLK2-D and TLK2-A on the gel. It is likely that the TLK2-A and TLK2-D mutations affect the phosphorylation of TLK2 at other sites, as under normal conditions, TLK2 has 12 phosphorylation sites (Fig. 7C). With more phosphorylation sites, the migration of TLK2-WT on the gel may be slower. Taken together, these findings suggest that calcium overload activates TLK2 via phosphorylation, increasing its kinase activity and leading to mitochondrial damage and lysosomal dysfunction, resulting in cell death.

### TLK2 KO rescued calcium overload induced DA neuron loss and α-synuclein-induced lesion in mice

**Fig. 8:**
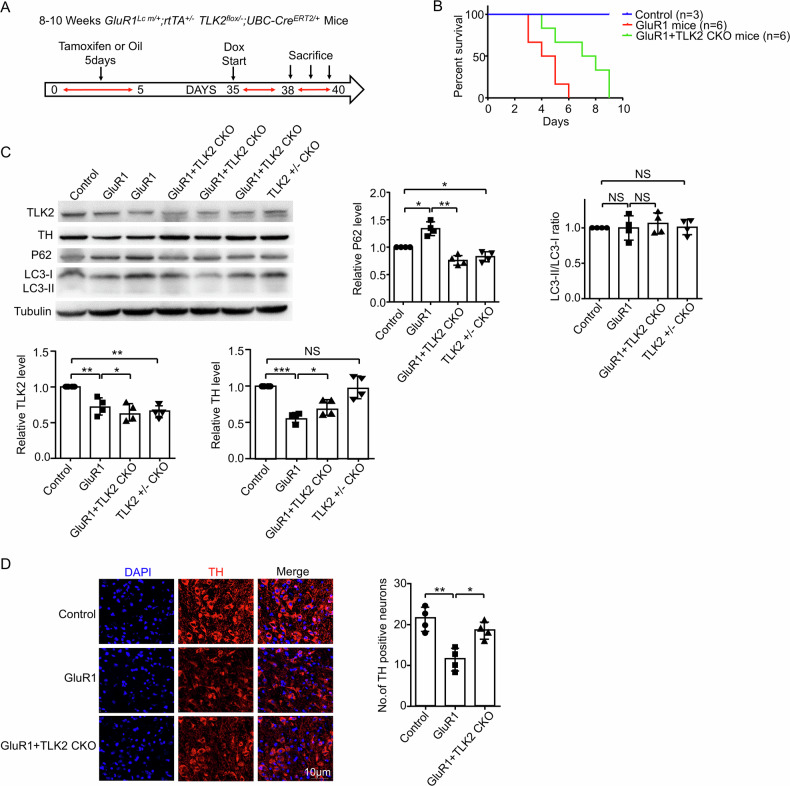
TLK2 KO rescued calcium overload induced DA neuron loss. **A** Schematic for experimental design in panels (**B**–**D**). Mice with 8-10 weeks-old were administered with tamoxifen (corn oil as the control) via intraperitoneal injection for 5 days to obtain heterologous TLK2 KO. One month later, doxycycline (dox) was given in drinking water to induce GluR1^Lc^ expression for 3–5 days. **B** Survival rate of the GluR1^Lc^, GluR1^Lc^ / TLK2 CKO and control mice. Control mice (*n* = 3 mice per group), GluR1^Lc^ and GluR1^Lc^ / TLK2 CKO (*n* = 6 mice per group). **C** Western blot of the tissue extracts from the GluR1^Lc^, GluR1^Lc^ / TLK2 CKO and control mice (*n* = 4 independent trials). Quantification of TLK2, TH, P62 and LC3-II/LC3-I ratio level. **D** Immunostaining images of the TH neurons (red) in the SNc, nuclei were stained with DAPI (blue) (*n* = 4 independent trials). Scale bar, 10 μm. Quantification of the number of the TH positive neurons.

To determine TLK2 effect on calcium-overload induced damage in vivo, we constructed a calcium overload model in mice. The glutamate receptor 1 Lurcher mutant (GluR1^Lc^) forms constitutively opened cation channel in a heterologous expression system [30]. We generated a transgenic mouse line which expresses GluR1^Lc^ conditionally (GluR1^Lc m/m^, “m” for GluR1^Lc^). Crossed with a doxycycline (dox), the progeny mice (GluR1^Lc m/+^; rtTA^+/-^) expresses GluR1^Lc^ ubiquitously when doxycycline is provided. We found that providing dox at normal concentration (2 mg/ml) in drinking water induced mouse lethality within one day. After dox dilution of 200 times (0.01 mg/ml), the GluR1^Lc^ mice could survive more than 3 days. Next, we obtained ubiquitous and conditional TLK2 knockout mice (TLK2^flox/flox^; UBC-Cre^ERT2/+^). Crossing these mice, we acquired the desired genotype (GluR1^Lc m/+^; rtTA^+/-^; TLK2^flox/-^; UBC-Cre^ERT2/+^). The experimental design was outlined (Fig. 8A). Mice with 8-10 weeks of age were administered with tamoxifen (with corn oil as a control) via intraperitoneal injection for 5 days to obtain heterologous TLK2 KO. One month later, dox (0.01 mg/ml) was provided in drinking water to induce the GluR1^Lc^ expression. We found that mice expressing GluR1^Lc^ started to die on day 3 after dox treatment, while mice with heterozygous TLK2 KO survived longer (Fig. 8B and supplementary video). Moreover, both TH level and TH positive neurons were reduced in the GluR1^Lc^ mice; and TLK2 KO alleviated this lesion (Fig. 8C, D). Similar to the case in *Drosophila*, the P62 protein level was increased in GluR1^Lc^ mice, and TLK2 KO reduced the P62 level (Fig. 8C).

**Fig. 9:**
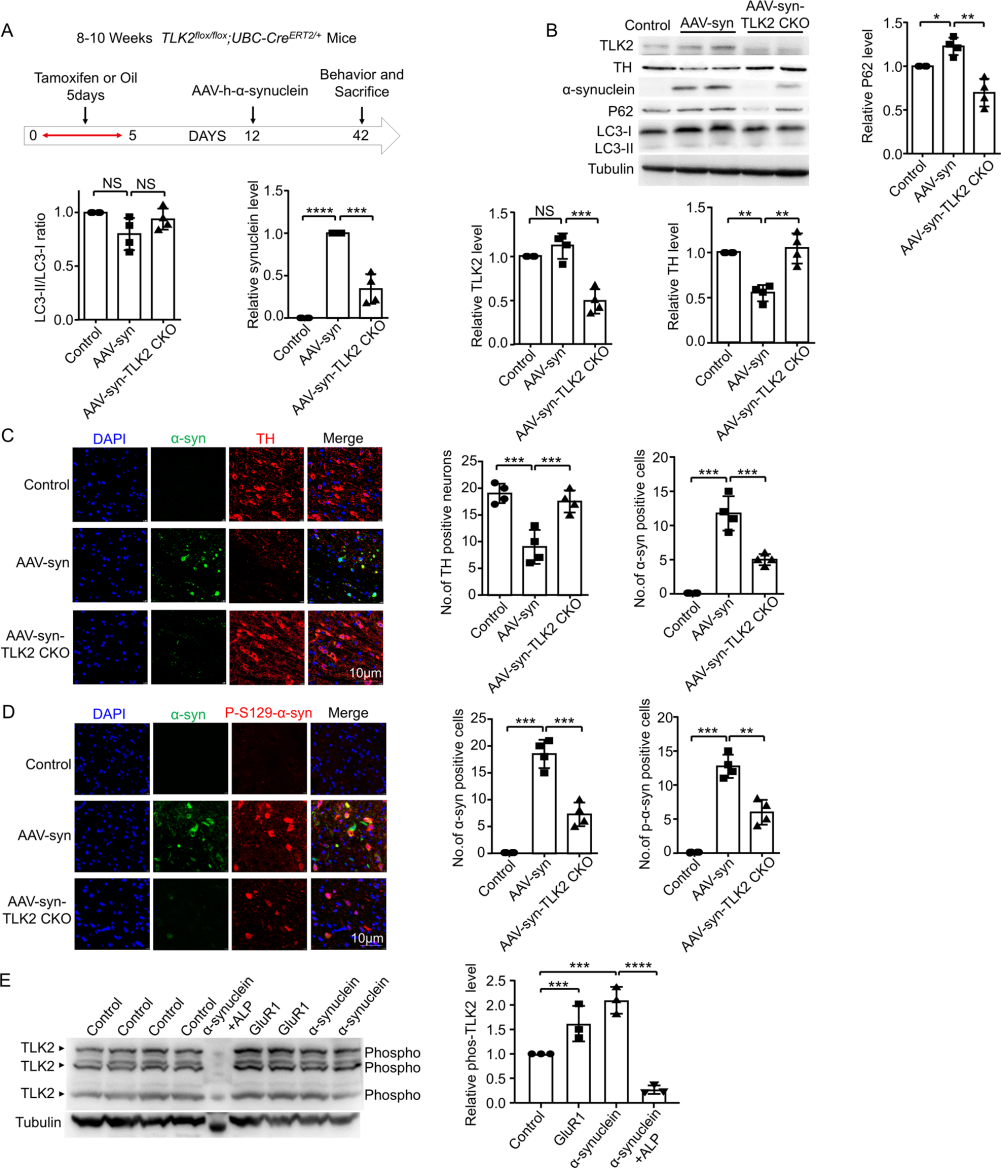
TLK2 KO rescued α-synuclein-induced lesion in mice. **A** Schematic experimental design in panels (**B**–**E**), homozygous TLK2 KO mice (TLK2^flox/flox^; UBC-Cre^ERT2/+^) at 8–10 weeks-old were obtained by administration tamoxifen (with corn oil as a control) via intraperitoneal injection. One week later, these mice were injected with AAV9-CMV-human-α-synuclein or PBS in the SNc. One month after the injection, the mice were tested for behavioral alternation. **B** Western blot of the tissue extracts from the AAV-syn, AAV-syn+TLK2 CKO, and control mice (*n* = 4 independent trials). Quantification of TLK2, TH, α-synuclein, P62 and LC3-II/LC3-I ratio level. **C** Immunostaining images of the TH neurons (red) in the SNc, nuclei were stained with DAPI (blue), α-synuclein were accompanied with GFP fluorescent protein (green) (*n* = 4 independent trials). Scale bar, 10 µm. Quantification of the number of the TH and α-synuclein positive cells. **D** Immunostaining images of the P-S129-α-synuclein (red) in the SNc, nuclei were stained with DAPI (blue), α-synuclein were accompanied with GFP fluorescent protein (green) (*n* = 4 independent trials). Scale bar, 10 μm. Quantification of the number of the P-S129-α-synuclein and α-synuclein positive cells. **E** Phos-tag western blot of the tissue extracts from the GluR1^Lc^, α-synuclein overexpression and control mice. ALP was used for dephosphorylation of protein serine, threonine and tyrosine residues. Tubulin was used as a loading control (*n* = 3 independent trials). Quantification of TLK2 phosphorylation level.

We also tested the effect of TLK2 KO on a-synuclein-induced lesions in mice. Injection of adeno-associated virus (AAV)-expressed human a-synuclein in the mouse midbrain induces neuropathological lesions in nigral DA neurons, including increased a-synuclein inclusions and progressive axonal degeneration [31]. The time frame of the experiment was outlined (Fig. 9A). Homozygous TLK2 KO mice (TLK2^flox/flox^; UBC-Cre^ERT2/+^) with 8–10 weeks of age were obtained by tamoxifen administration (with corn oil as a control) via intraperitoneal injection. One week later, these mice were injected with AAV9-CMV-human-a-synuclein or PBS into the right side of the substantia nigra pars compacta (SNc). One month after the injection, the mice were tested for behavioral abnormalities. In both rotarod and open field tests, the mice expressing a-synuclein performed worse than the control group, and TLK2 CKO mice showed better performance (Supplementary Fig. S5A, B). Compared to the left side of the brain (control), the lesion on the right side of the brain showed reduced TH protein levels and increased a-synuclein and P62 protein levels, which were alleviated by TLK2 CKO (Fig. 9B, C). Moreover, the sagittal section of the mouse brain showed the lesion side (the left side of the micrograph) with increased a-synuclein accumulation associated with reduced TH-positive staining, which was alleviated in the TLK2 CKO background (Supplementary Fig. S5C). Ser129-phosphorylated a-synuclein is considered a marker of a-synuclein neuropathology [32]. Mice overexpressing a-synuclein showed accumulation of Ser129-phosphorylated a-synuclein on the injection side, and TLK2 KO significantly reduced this increase (Fig. 9D). Strikingly, increased TLK2 phosphorylation was detected in the brains of GluR1^Lc^ and a-synuclein overexpression mice (Fig. 9E), suggesting that TLK2 is activated under these pathological conditions. Collectively, these results indicate that TLK2 activation plays a key cytotoxic role in a-synucleinopathies.

## Discussion

### Disruption of calcium homeostasis is a key cytotoxic event of a-synucleinopathy

Mounting evidence demonstrates that PD-related mutations or environmental risk factors, such as aging and exposure to neurotoxins, are associated with defective calcium homeostasis through a wide variety of mechanisms [3]. Because calcium signaling plays a prominent role in multiple aspects of neuronal physiology, loss of calcium homeostasis is clearly pathogenic, especially its effect on mitochondrial dysfunction and disruption of the autophagy-lysosome pathway [33, 34].

As a hallmark of PD, the accumulation of a-synuclein induces cytotoxicity through diverse mechanisms, including defective calcium homeostasis, ER, Golgi, nuclear, mitochondrial and autophagy-lysosomal dysfunctions [15]. The shared spectrum of cytotoxicity induced by calcium overload and a-synuclein accumulation prompted us to search for their common regulators. Here, we studied the calcium overload model *MC16* in *Drosophila*. After induction of the leaky cation channel, fly muscle cells showed slow and progressive damage in the first 3–7 days after eclosion. This suggests that *MC16* induces a slow onset of calcium overload, which is significantly milder than that in the neuron necrosis model as reported previously [35]. Consistent with mild calcium overload stress, cell death in the muscle cells of *MC16* did not result in necrosis because of negative PI staining. In addition, our data demonstrated that a-synuclein overexpression (*DaGS* > *SNCA*) in *Drosophila* could induce calcium overload, autophagy and mitochondrial dysfunction, and DA neuron death, and these deficits could be rescued by TLK RNAi. Consequently, we hypothesize that calcium overload constitutes a critical cytotoxic event in a-synucleinopathy.

### Calcium overload inhibits the lysosomal pathway and impairs mitochondrial function by TLK/TLK2

In calcium overload model, we found calcium overload interferes with the normal functioning of lysosomes, hindering the cell’s ability to degrade and recycle damaged components. By RNA sequencing, we found TLK2 RNAi could rescue several lysosomal functional genes, including lysosomal acidification (CG7997, CtsB and Gba1b) and hydrolyzation (VhaSFD) genes, which were inhibited by calcium overload. Besides, lysosome-autophagy function was activated by TLK2 KO and inhibited by TLK2 overexpression. Therefore, we proposed that TLK (or TLK2) could regulate the lysosomal functional genes by transcription.

As we all known, cytosolic calcium overload almost result in mitochondrial calcium overload. While this response may damage mitochondrial function, leading to increased reactive oxidative species (ROS) production, ATP synthesis inhibition and mitochondrial permeability transition pore (mPTP) opening. In this paper, we found calcium overload resulted in mitochondrial transmembrane potential decreasing and mitochondrial fragmentation. However, downregulating of TLK (or TLK2) rescued it, and overexpression of TLK (or TLK2) enhanced it, indicating calcium overload may regulate the mitochondrial function by TLK (or TLK2).

These findings suggest a complex interplay between calcium homeostasis, lysosomal and mitochondrial function in the pathogenesis of a-synucleinopathy. The disruption of lysosomal function due to calcium overload may lead to the accumulation of toxic proteins, including α-synuclein, further exacerbating cellular stress. Moreover, the impairment of mitochondrial function could result in decreased energy production and increased oxidative stress, potentially contributing to the progressive neurodegeneration observed in a-synucleinopathy. These findings highlight the importance of maintaining calcium homeostasis for proper cellular function and suggest potential therapeutic targets for a-synucleinopathy. Future research should focus on developing strategies to restore calcium balance and enhance lysosomal and mitochondrial function in affected cells. Additionally, investigating the specific mechanisms by which calcium overload disrupts these cellular processes may provide valuable insights into the progression of a-synucleinopathy and other neurodegenerative disorders.

### The TLK2 inhibitor may represent a potential therapeutic target for PD

The normal functions of TLK1 and TLK2 are involved in chromatin assembly, DNA damage response and transcription [36, 37]. TLK2 haploinsufficiency causes distinct neurodevelopmental disorders, and TLK upregulation drives cancer cell proliferation [38, 39]. In this study, we found that TLK2 heterozygous KO could reduce calcium overload triggered neurodegeneration, and TLK2 conditional homozygous KO could rescue a-synuclein-induced DA neuron death. We observed that TLK2 KO in adult stage did not generate observable behavioral defects in mice, such as appearance, mobility and weight gain, suggesting a safety profile to target TLK2 in the adult stage.

In calcium overload-induced cell death, we found that calcium overload could activate TLK/TLK2 by enhancing TLK/TLK2 phosphorylation, increasing its kinase activity. We detected 15 serine/threonine sites of TLK2 that were phosphorylated when purified TLK2 was treated with cell lysate undergoing calcium overload. In addition, our results showed that promazine hydrochloride (PMZ), a TLK2 kinase inhibitor, rescued a-synuclein-induced lysosomal decline in SK-N-SH cells. However, PMZ showed pronounced side effects as an antipsychotic drug and was deprescribed [40]. We are screening the new TLK2 kinase inhibitor, and we find three potential drugs which have the same efficacy as PMZ. A small molecule library (over 2000 active small molecules) was screened using a cellular calcium overload model. The identification of the most specific TLK2 inhibitors is desirable in the future.

These potential drugs could offer a promising alternative to PMZ, potentially mitigating the severe side effects associated with antipsychotic medications. Further investigation into their specificity, efficacy, and safety profiles is crucial to determine their viability as therapeutic agents in neurodegenerative disorders.

### Limitations of this study

We have not determined the threshold a-synuclein level to induce calcium overload. We also do not know the threshold of calcium overload to trigger cell death in different cell types and whether TLK2 phosphorylation is sufficient to induce cell death. Moreover, the mechanism of cell death induced by *Drosophila* TLK and mammalian TLK2, with the roles of lysosome and mitochondria in the cell death process, is unknown. Understanding these questions is important to elucidate the pathology of calcium overload in the context of related neurodegenerative diseases.

In summary, this study demonstrates that calcium overload plays a critical role in the cytotoxicity associated with a-synucleinopathy, leading to mitochondrial damage, lysosomal dysfunction, and cell death. Genetic screening in a *Drosophila* model of calcium overload identified Tousled-like kinase (TLK) as a key regulator of these pathological processes. Loss of TLK function mitigated the defects induced by both calcium overload and a-synuclein overexpression in *Drosophila*. In mammalian cells and mice, calcium overload activated TLK2 (the homologue of *Drosophila* TLK) by enhancing its phosphorylation, which increased its kinase activity. TLK2 knockout mice exhibited rescue of the cytotoxicity induced by calcium overload and a-synuclein overexpression. These findings suggest that TLK2 activation by calcium overload is a pivotal step in the progression of Parkinson’s disease, providing a potential link between calcium overload and the subsequent mitochondrial and lysosomal dysfunction observed in the disease.

## Methods

**Key resources table**

**Table:** 

Reagent or resource	Source	Identifier
Antibodies		
β-tubulin	TransGen	Cat# HC101, RRID: AB_2893358
α-synuclein	BD Biosciences	Cat# 610787, RRID: AB_398108
TH	Millipore	Cat# AB152, RRID: AB_390204
Ref(2)p	Abcam	Cat# ab178440, RRID: N/A
GABARAP	Cell Signaling Technology	Cat# 13733S, RRID: AB_2798306
P62	Abcam	Cat# ab56416, RRID: AB_945626
LC3A/B	Cell Signaling Technology	Cat# 12741S, RRID: AB_2617131
TLK2	Proteintech	Cat# 13979-1-AP, RRID: AB_2203887
P-S129-α-Syn	Biolegend	Cat# 825701, RRID: AB_2564891
TLK1	Cell Signaling Technology	Cat# 4125S, RRID: AB_2203885
Lamp2a	Abcam	Cat# ab18528, RRID:AB_775981
Bacterial and virus strains		
Trans1-T1	TransGen	CD501-02
BL21(DE3)	TransGen	CD601-02
LC3-mRFP-GFP adenovirus	Hanbio Biotechnology	N/A
AAV9-CMV-h-α-synuclein	Hanbio Biotechnology	N/A
Chemicals, peptides, and recombinant proteins		
Goat anti-mouse IgG-HRP	Gene-Protein Link	P03S01S
Goat anti-rabbit IgG-HRP	Gene-Protein Link	P03S02S
Fluo4-AM	Invitrogen	F14201
Fura2-AM	Invitrogen	F1221
Pluronic™ F-127	Invitrogen	P3000MP
TUNEL	Roche	12156792910
F-actin	Invitrogen	A12379
TMRM	Invitrogen	T668
Secondary antibody: Alexa Fluor 488 Goat anti-rabbit IgG secondary (H + L)	Invitrogen	A11034
Secondary antibody: Alexa Fluor 488 Goat anti-mouse IgG secondary (H + L)	Invitrogen	A11029
Secondary antibody: Alexa Fluor 568 Goat anti-rabbit IgG secondary (H + L)	Invitrogen	A11036
Secondary antibody: Alexa Fluor 568 Goat anti-mouse IgG secondary (H + L)	Invitrogen	A11031
LysoTracker	Invitrogen	L7528
Mito Tracker	Invitrogen	M7512
DAPI	Beyotime	C1005
Immobilon Western HRP Substrate	Millipore	WBKLS0500
Trizol	Invitrogen	15596026
DMEM	Gibco	11995081
FBS	Gibco	10099-141c
PS	Gibco	15140-122
Lipo8000	Beyotime	C0533
Ionomycin	Sigma	I3909
Doxycycline	Sigma	D9891
Tamoxifen	Sigma	T5648
PI	Sigma	P4170
Critical commercial assays		
RNA extract kit	Beyotime	R0027
GST-tag Protein Purification Kit	Beyotime	P2262
Cell Counting Kit-8	Beyotime	C0038
cDNA Synthesis Super Mix	TransGen	AH301
SYBR™ Green Mater Mix	Applied Biosystems	A25742
Experimental models: Cell lines		
Hela	ATCC	CCL-2
SK-N-SH	ATCC	HTB-11
293T	ATCC	CRL-3216
MES23.5	FENGHUISHENGWU	CL0466-1
Experimental models: Organisms/strains		
*Drosophila: w* ^*1118*^	Bloomington Drosophila Stock Center	BL# 3605
*Drosophila: Mhc-gal4*	Bloomington Drosophila Stock Center	BL# 38464
*Drosophila: UAS-SNCA*	Bloomington Drosophila Stock Center	BL# 51376
*Drosophila: UAS-TLK*	Bloomington Drosophila Stock Center	BL# 29964
*Drosophila: UAS-GFP*	Bloomington Drosophila Stock Center	BL# 5431
*Drosophila: UAS-ER-RFP*	Bloomington Drosophila Stock Center	BL# 30909
*Drosophila: UAS-mito-RFP*	Bloomington Drosophila Stock Center	BL# 93056
*Drosophila: Daughterless-gal4*	Tsinghua Fly Center	TB00153
*Drosophila: TLK RNAi*	Tsinghua Fly Center	THU1326
*Drosophila: TLK RNAi*	Tsinghua Fly Center	THU2010
*Drosophila: REPTOR RNAi*	Tsinghua Fly Center	THU3990
*Drosophila: TRiP control*	Tsinghua Fly Center	TB00072
*Drosophila: UAS-C16*	This paper	N/A
Mouse: GluR1^Lc^	Shanghai Model Organisms Center	N/A
Mouse: rtTA^+/+^	Shanghai Model Organisms Center	N/A
Mouse: TLK2^flox/flox^	Shanghai Model Organisms Center	N/A
Mouse: UBC-Cre^ERT2/+^	Shanghai Model Organisms Center	N/A
Oligonucleotides		
Primers for qPCR analysis, see Supplementary Table S3	N/A	N/A
Software and algorithms		
GraphPad Prism 8	GraphPad Software	https://www.graphpad.com:443/scientific-software/prism/, RRID:SCR_002798
ImageJ Fiji v2.0.0-rc-68/1.52 h	(Schindelin et al., 2012)	https://fiji.sc/; RRID: SCR_002285
The FUSION FX Spectra software	Vilber	https://www.vilber.com/fusion-fx-spectra/
QuantStudio™ Real-Time PCR Software	Thermo Fisher Scientific	https://www.thermofisher.com/jp/ja/home/global/forms/life-science/quantstudio-6-7-flex-software.html
Leica SP8 softwore	Leica	https://www.leica-microsystems.com/

### *Drosophila* stocks and maintenance

*Drosophila* stocks were maintained under standard conditions at 25 °C on agar, cornmeal and yeast food. The flies included *w*^*1118*^*, Mhc-gal4, UAS-SNCA, UAS-TLK, UAS-GFP, UAS-ER-RFP* and *UAS-mito-RFP* were purchased from Bloomington *Drosophila* Stock Center; *Daughterless-gal4, TLK RNAi, REPTOR RNAi, TRiP control* and all other TRiP lines were obtained from Tsinghua Fly Center. The *UAS-C16* and *UAS-REPTOR* transgene were generated under the *w*^*1118*^ background by P-element insertion in our laboratory. For *DaGS* > *SNCA* flies, 20 flies were collected and placed in one tube at 1–3 days, 500 μM RU486 (Mifepristone; MedChemExpress #HY-13683) was mixed in the standard food, the fly vials were changed to new vials every two days. For *MC16* fly survival, 1–3 days-old flies were collected and transferred to new vials every two days. The dead flies were recorded until all flies were dead.

### Western blot

Tissues or cells were lysed in RIPA buffer (50 mM Tris, 150 mM NaCl, 1% Triton X-100, 1% sodium deoxycholate and 0.1% SDS) supplemented with protease inhibitor cocktail (Roche #11697498001) and PMSF (Beyotime #ST507) for 30 min on ice. Then the lysis was sonicated for 1 min with 40 w 3 s on/3 s off on ice and centrifugation at 12,000 rpm 4 °C for 15 min. Supernatants were collected and boiled with SDS loading buffer at 95 °C for 5 min. 20–30 μg protein from cell lysate or tissue homogenate were loaded on 12% SDS-PAGE gels. Transferring the protein from the gel to the PVDF membrane (Millipore #ISEQ00010). Membranes were blocked with 5% milk for one hour at room temperature and incubated with appropriate dilutions of primary antibody overnight at 4 °C. Washing the membrane 3 times for 5 min each with TBST (TBS containing 0.1% Tween20), and then incubating with HRP secondary antibody (1:10000) for one hour at room temperature. Acquiring image using HRP substrate (Millipore #WBKLS0500) for chemiluminescence with VILBER Fusion FX SPECTRA equipment.

### Phos-tag Western blot

Mice brain tissues were quickly homogenized in ice-cold RIPA buffer supplemented with protease inhibitor cocktail, PMSF and phosphatase inhibitor (Beyotime #P1082) for 30 min on ice. Then the lysis was centrifugation at 12,000 rpm 4 °C for 15 min, supernatants were collected, part of the supernatants were treated with alkaline phosphatase (Beyotime #D7027) and boiled with SDS loading buffer at 95 °C for 5 min. 40 μmol/L MnCl_2_ and 20 μmol/L phos-tag (Wako #304-93521) were added to the SDS-PAGE (8%). After the electrophoresis, the phos-tag gels were incubating with transfer buffer containing 5 mM EDTA for 20 min twice to remove the divalent cations [41]. Washing with transfer buffer for 10 min once. Transferring the protein from the gel to the PVDF membrane. The next blocking and antibody incubation were the same as the normal western blot procedures.

### Immunofluorescence staining

Mice were anesthetized with pentobarbital sodium (80 mg/kg) and perfused with 0.9% saline followed by 4% paraformaldehyde (PFA). The brains were removed and placed in 4% PFA overnight, then transferred to 10%, 20% and 30% sucrose until the brains dropped to the bottom. The brains were embedded in OCT (SAKURA #4583) and cut into 10 μm thick coronal slices. The *Drosophila* brain or indirect flight muscle were dissected in HL3 buffer (70 mM NaCl, 5 mM KCl, 0.2 mM CaCl2, 20 mM MgCl2, 10 mM NaHCO3, 5 mM trehalose, 115 mM sucrose, and 5 mM HEPES). Brain slices, *Drosophila* tissues or cells were fixed with 4% PFA for 15 min at room temperature, and then incubated three times for 5 min each with PBST (PBS containing 0.2% Triton X-100) for permeabilization, followed by blocking with 5% BSA in PBST for 30 min at room temperature. Then incubation with primary antibody in the blocking solution overnight at 4 °C. Washing with PBS three times for 5 min each with gentle shaking. Incubating with secondary antibody (1:200) for 1 h (light protected) and repeat washing, optional staining with DAPI solution (Beyotime #C1005) for 5 min and rinsing with PBS. Mounting coverslip with mounting medium (abcam #ab104135). Acquiring images with Leica SP8 confocal microscope.

### Quantitative real-time PCR

Total RNAs were extracted according to the manufacturer’s protocol (Beyotime #R0027). 5 μg of total RNA were reversed using the TransScript® II First-Strand cDNA Synthesis SuperMix kit (TransGen #AH301) based on the manufacturer’s instructions. The final volume of qRT-PCR reaction mixer was 10 μl containing 5 μl PowerUp SYBR Green Master Mix (Applied Biosystems #A25742), 1 μl diluted cDNA sample (1:1), 0.5 μl forward primer, 0.5 μl reverse primer and 3 μl Nuclease-free water. The quantification of target genes was calculated by ΔCt method with Applied Biosystems QuantStudio™ Real-Time PCR System. Actin was used as a reference.

### Fluo4-AM staining

Fly brains or muscles were dissected in HL3 buffer, and incubated with 5 μM Fluo4-AM (Invitrogen #F14201) and 0.02% Pluronic F-127 (Invitrogen #P3000MP) at 25 °C for 30 min in the dark. Then washing with HL3 buffer three times for 10 min each. Measuring fluorescence using Leica SP8 confocal microscope for excitation at 494 nm and emission at 516 nm [42].

### Fura2-AM staining

Fly indirect flight muscles were dissected in HL3 buffer, and incubated with 1 μM Fura2-AM (Invitrogen #F1221) and 0.02% Pluronic F-127 (Invitrogen #P3000MP) at 25 °C for 30 min in the dark. Then washing with HL3 buffer three times for 10 min each. Measuring fluorescence using IONOPTIXBIOION-1.3 confocal microscope for excitation at 340 nm and 380 nm and the fluorescence intensities detected at ~510 nm by WinFluor software.

### Histology of *Drosophila* adult thorax or brain

*Drosophila* indirect flight muscle (IFM) were dissected in HL3 buffer, GFP, mito-RFP and ER-RFP were imaged by Leica SP8 confocal microscope respectively. Fly tissues were incubated with 1 μM lysotracker (Invitrogen #L7528) at 25 °C for 10 min in the dark. Then washing with HL3 buffer three times for 10 min each. For TMRM (Invitrogen #T668) staining, the experimental procedures were similar to the lysotracker staining.

### TUNEL staining

Fly indirect flight muscle (IFM) were dissected and fixed with 4% PFA for 15 min at room temperature, and then incubated three times for 5 min each with PBST (PBS containing 0.2% Triton X-100), followed by adding 10 μg/ml proteinase K solution diluted with PBS for 10 min at 56 °C. Washing with PBST three times for 5 min each. The TUNEL (Roche #12156792910) reaction mixture were incubated for 2 h at 37 °C, and protected from light. Washing with PBST three times for 5 min each. Then F-actin (Invitrogen #A12379) were dissolved into DMSO to make a 1000x stock solution, diluting this stock solution 1:1000 in PBS and staining for one hour at room temperature, Washing with PBS three times for 5 min each. Acquiring images with Leica SP8 confocal microscope.

### Transmission electron microscopy

The fly muscles or cells were fixed with 2.5% glutaraldehyde in 0.1 M PB buffer for 4 h at 4 °C. Washing with 0.1 M PB 3 times for 10 min each. Then sample were prepared for imaging according to the standard protocol and observed using a transmission electron microscopy (HT7700).

### RNA sequencing

Total RNAs were extracted using Trizol (Invitrogen #15596026) reagent based on the manufacturer’s instructions, the integrity and quantity of RNA sample using agarose gel electrophoresis and NanoDrop ND-1000 (Thermo Scientific, USA). RNA sample were sequenced by Illumina HiSeq 2500 instrument, differentially expressed genes were analyzed.

### Data deposition

The data has been deposited on Aliyun Dbank (link: https://www.alipan.com/s/AqPhqENaUyv,Code:97hx).

### Generation of TLK1 and TLK2 knockout in Hela cells

Guide RNAs were designed by CRISPOR (http://crispor.tefor.net/crispor.py), TLK1 gRNA (5’-AAAGTATTGGGGGA-3’) was recognized the Exon5 and TLK2 gRNA (5’-ATATCTCTAGGCAACAGGAA-3’) was recognized the Exon12. gRNAs were cloned into Cas9 vector-PX459 and then transfected to the Hela cells with Lipo8000 (Beyotime #C0533). After 48 h transfection, 3 μg/ml puromycin were added to the fresh medium, cells were cultured for 24 h to eliminate the cells without recombinant plasmid. Then transfer the survival cells into a 96-well plate to expand single clones for approximately 3 weeks, genomic DNA were extracted and sequencing after PCR amplification using the following primers TLK1: F: TCACTTTGTCAGCGTGGTCA, R: CCACGGTCTGACTGCAAAAA; TLK2: F: CGTGTAGAGTAGTAATGGCTCCC, R: ACCAGGAGTGGCCAAAAGTC. TLK1 and TLK2 knock out cells were confirmed by western blot.

### Cell viability assay

Cell viability was assessed using the Cell Counting Kit-8 (beyotime, C0038). Cells were seeded in 96-well plates the day before treatment. Hela WT, TLK2 KO or TLK2 overexpression cells were cultured with or without treatment with ionomycin (1 μM for 24 h). Add 10 μL of CCK-8 solution to each well. Continue incubating for 2 h. Measure the absorbance at 450 nm.

### Live mitochondrial morphology imaging

Cells were incubated with 200 nM Mito Tracker for 30 min, finally washed 3 times with PBS. Images were acquired using Leica SP8 laser scanning confocal microscope equipped with a 63 NA oil objective as Z stacks, with identical imaging parameters among different phenotypes in a blinded fashion.

### LC3-mRFP-GFP adenovirus transfection

Hela cells were cultured in DMEM medium (Gibco #11995081) supplemented with 10% FBS (Gibco #10099-141c), 1% streptomycin and penicillin (Gibco #15140-122). When cell density was between 50% and 70% in confocal dish, LC3-mRFP-GFP adenovirus (Hanbio Biotechnology) were added to 1 ml medium at multiplicity of infection (MOI) of 10 [43]. After 6–8 h transfection, changing the medium and further culturing for 24 h, cells were imaged with Leica SP8 confocal microscope.

### GST pull down assay

For TLK2 purification, TLK2 cDNA was cloned into the PGEX-6P-1 vector containing a GST tag. This construct was expressed into the BL21 chemically competent cells (TransGen # CD701), and the induced condition was 0.5 mM IPTG, 180 rpm rotation for 20 h at 20 °C. GST-TLK2 was purified by GST-tag Protein Purification Kit (Beyotime #P2262) according to the manufacturer’s protocol.

Hela cells were treated with 1 μM ionomycin (Sigma # I3909) for 3 h when the cells density reached to 70% confluence. Then cells were harvested and lysed in lysis buffer (10 mM Tris, 150 mM NaCl, 2 mM EDTA, 0.5% TritonX-100). After centrifugation at 12,000 rpm 4 °C for 15 min, supernatants were collected. Mixing 3 μg GST-TLK2 purification protein and 200 μg cell lysate, rotation at 4 °C overnight. After rotation, GST-TLK2 were pulled down by Glutathione Sepharose 4B (GE Healthcare #17-0756-01) for 1 h rotation at room temperature. Washing with lysis buffer 6 times at 2000 rpm 4 °C for 5 min each. Interactions between GST-TLK2 and beads were disrupted by 4× loading buffer at 95 °C for 5 min. The final results were obtained by SDS-PAGE, phos-tag SDS-PAGE, Coomassie Blue staining and mass spectrometry, or TLK2 protein were cut by PreScission Protease (Beyotime #P2302) overnight at 4 °C.

### TLK2 kinase assay

Standard kinase assay was performed for 15 min at room temperature in 50 μl of kinase buffer (10 mM Tris pH 7.5, 50 mM KCl, 10 mM MgCl2, 1 mM DTT) supplemented with 1 μl ATP (0.2 mM) and with 1 μg TLK2 purification proteins and 20 μg substrates. MBP (Millipore #13–110), PGK1 or CREBRF were the substrates used for the kinase assays [28].

### GluR1^Lc^ transgenic mice

All the mice were housed in the animal center of Capital Medical University. The animal studies were approved by the Institutional Animal Care and Use Committee, Capital Medical University, Beijing, China (approved number: AEEI-2015-156). GluR1^Lc^ mice were purchased from Shanghai Model Organisms Center. The SA-polyA-Insulator-Insulator-TRE-miniCMVpromoter-GluR1(Mut)-wpre-pA-Insulator-Insulator-FRT-PGK-Neo-pA-FRT was inserted at the Gt(ROSA)26Sor (ENSMUSG00000086429) locus by In-Fusion cloning. The GluR1^Lc m/m^ (ENSG00000155511) transgenic mice were crossed with rtTA^+/+^ mice, doxycycline (Sigma #D9891) was given in drinking water to induce GluR1^Lc^ expression.

### TLK2 knockout mice

TLK2 knockout mice were also purchased from Shanghai Model Organisms Center. The Cre-lox system was used for the deletion of TLK2 gene (ENSG00000146872). TLK2^flox/flox^ mice were generated by CRISPR-Cas9 technology which targeted exon4. After mating them with UBC-Cre^ERT2/+^ mice, TLK2 genes could be knocked out ubiquitously by tamoxifen (Sigma #T5648) via intraperitoneal injection in 75 mg/kg body weight once every 24 h for a total of 5 consecutive days. After the conditional knockout of TLK2, the mice appeared normal, including appearance, weight gain, mobility, and eating and drinking.

### Stereotactic injection of virus

Male, 8–10 weeks old, C57BL/6 mice were anesthetized with pentobarbital sodium (80 mg/kg) and placed in a stereotaxic frame (RWD, china). AAV9-CMV-h-α-synuclein (Hanbio Biotechnology) was injected into in the appropriate location (AP: −3.0 mm; ML: −1.3 mm; DV: –4.5 mm) for the substantia nigra (SN) at the right of the skull [44]. 1 μl of AAV9-CMV-h-α-synuclein (concentration: 1.5 ×10^12^ vg/ml) or PBS were injected at a rate of 0.1 μl/min with a 10 μl Hamilton syringe. The needle was left in the place for a further 5 min after injection to avoid leakage. After the surgery, mice were placed on a warm electric blanket for recovery.

### Mice behavioral tests

In the open field test, mice were acclimated to the experimental room in their home cages for 30 min to minimize stress and then were placed into the open field cage (50 × 50 × 50 cm). After 1 min of adaptation, the mice were allowed to explore the arena for 5 min. After the 5 min recording by SMART 3.0 software (RWD, china), mice were returned to their home cages and the arena was cleaned with 70% ethanol [45].

In the rotarod test, mice were placed on an accelerating rotarod at speeds between 5 and 45 rpm for a maximum of 5 min. The animals were tested for 3 days with 2 trials per day, the latency to fall was recorded and the last 2 trials of the 3^rd^ day were analyzed for locomotor abilities [46].

### Statistical analyses

For tests to quantify animal behavior and for sample collections, double blind experiments were performed. The animal studies were randomly assigned to experimental groups. In each quantification, data points were from individual animal or groups of flies (10–50 flies for one data point). Results were presented as mean ± SD. The number of animals studied was based on our previous experience and preliminary investigations. We did not exclude any data from this study. In all figures, data points are from individual mice and rats or groups of flies. Statistical significance for comparisons between data sets was primarily done with nonparametric tests. For comparison of more than two groups, significance was determined using a one-way ANOVA with Tukey’s post hoc test. Kaplan–Meier tests were used under the log-rank algorithm for survival analysis. Statistical analyses were performed using GraphPad Prism software. *P* value of <0.05 was considered significant (*P < 0.05, **P < 0.01, ***P < 0.001 and ****P < 0.0001), with “ns” denoting non-significant outcomes (*P* ≥ 0.05).

## Supplementary information

Supplementary Figure Legends

Figure S1

Figure S2

Figure S3

Figure S4

Figure S5

Table S1

Table S2

original data

VIDEO

## Data Availability

The materials used in this study are available upon request.
