# Supplementary material for: Calcium overload induced mitochondrial and lysosomal dysfunction is regulated by Tousled-like kinase in a-synucleinopathy
Source: Cell Death Dis. 2026 Jan 8;17(1):10. doi: 10.1038/s41419-025-08213-8 (PMC12783599; doi:10.1038/s41419-025-08213-8)
Supplement: Supplementary file 1 — Supplementary Figure Legends [file 41419_2025_8213_MOESM1_ESM.docx]

**Supplementary Figure Legends**

**Figure S1. Characterization of *MC16* flies; *TLK RNAi* effects of *MC16* flies and α- synuclein overexpression flies**

(**A**) Calcium overload level was measured by Fura2-AM (1 μM) calcium indicator. Intracellular Ca^2+^ level could be estimated by Fura2 340/380 ratio (n=3 independent trials). (**B**) The lifespan changes of *Mhc-gal4* (wild type control) and *MC16* flies. 80-100 flies were tested for each trial (n=3 independent trials). (**C**) Quantitative RT-PCR to analyze TLK mRNA expression from *Mhc-gal4/+, Mhc-gal4/TLK RNAi* (THU1326) and *Mhc-gal4/TLK RNAi-1* (THU2010) flies. 20 flies were tested for each trial (n=3 independent trials). (**D**) Quantitative RT-PCR to analyze C16 mRNA level from *MC16/+, MC16/TLK RNAi* and *MC16/TLK RNAi-1*. 20 flies were tested for each trial (n=3 independent trials). (**E**) TUNEL (red) staining of the indirect flight muscle, muscles were stained with F-actin (green). Images are representative of six flies with the indicated genotypes. Scale bar, 10 μm. (**F**) Propidium Iodide (PI) staining of the *Mhc-gal4* and *MC16* flies, nuclei were stained with DAPI (blue). Images are representative of six flies with the indicated genotypes. Scale bar, 5 μm. (**G**) Calcium overload level was measured by Fluo4-AM (5 μM, green) calcium indicator (n=10 tissues from 10 flies). Scale bar, 10 μm.

**Figure S2. *TLK RNAi* rescued the cell damage induced by a-synuclein overexpression**

(**A**) Calcium overload level was measured by Fluo4-AM (5 μM, green) calcium indicator of the brain and the muscle in 20 days-old, the relative intensity was analyzed by F/F0, F represented the fluorescence intensity (n=10 tissues from 10 flies). Brain Scale bar, 2.5 μm, Muscle Scale bar, 5 μm. (**B**) Western blot of the tissue extracts from the whole body in 20 days-old. Quantification of TH and α-synuclein level. 20 flies were tested for each trial (n=3 independent trials). (**C**) Immunostaining images of the TH neurons (green) in *DaGS>SNCA* fly brain PPL1 cluster. Scale bar, 2.5 μm. 5 flies were tested for each trial (n=3 independent trials). Quantification of the number of the TH positive neurons. (**D**) Calcium overload level was measured by Fluo4-AM (5 μM, green) calcium indicator of the brain and the muscle in 30 days-old (n=10 tissues from 10 flies). Brain Scale bar, 2.5 μm, Muscle Scale bar, 5 μm. (**E**) Quantitative RT-PCR analysis of SNCA mRNA expression from *DaGS>SNCA/+* and *DaGS>SNCA/TLK RNAi* flies. 20 flies were tested for each trial (n=3 independent trials). (**F**) Live imaging of the ER morphology of the indirect flight muscle. The ER is labeled with *UAS-ER-RFP* driven by *Mhc-gal4*. Images are representative of six flies with the indicated genotypes. Scale bar, 5 µm.

**Figure S3. TLK1 and TLK2 KO effect on transcriptional level of lysosomal genes and autophagy-lysosome function**

(**A**) Quantitative RT-PCR analysis of five lysosomal genes mRNA expression from Hela WT, TLK2 KO, WT+ starved in Earle's starvation buffer (EBSS) and TLK2 KO+EBSS cells. EBSS is a powerful inducer of autophagy in cultured cells (n=3 independent trials). (**B**) Quantitative RT-PCR analysis of five lysosomal genes mRNA level from Hela WT and TLK1 KO cells (n=3 independent trials). (**C**) Western blot of the extracts from the α-synuclein overexpression, or α-synuclein overexpression plus PMZ (TLK kinase inhibitor) treatment, or control cells (n=3 independent trials). α-synuclein, P62 and LC3-II/LC3-I ratio is quantified. (**D**) Lysotracker (red) staining in cells overexpressed α-synuclein, or α- synuclein overexpression plus PMZ treatment, or the control cells (n=20 independent trials). Scale bar, 10 µm. Quantification of the number of lysotracker positive vesicles. (**E**) Western blot of the extracts from Hela WT and TLK1 KO cells (n=3 independent trials). Quantification of P62 level and LC3-II/LC3-I ratio. (**F**) Immunostaining images of the P62 protein (red) in the P62 overexpression Hela cells, nuclei were stained with DAPI (blue) (n=3 independent trials). Scale bar, 5 µm.

**Figure S4.** **The conserved and divergent features of TLK in *Drosophila* and TLK2 in mammals.**

The protein sequences of TLK in drosophila is1266 aa, and the TLK2 in mammals is 772aa. The percent identity matrix is 52.33%. we compared the protein sequences between TLK in *Drosophila* and TLK2 in mammals.

**Figure S5. Effect of TLK2 KO on α-synuclein induced DA neuron loss and lethality of GluR1Lc mice**

(**A**) Rotarod behavioral tests of AAV-syn, AAV-syn+TLK2 CKO, and control mice (n=6 mice for each genotype). Quantification of the time of the latency to fall. (**B**) Open field behavioral tests of AAV-syn, AAV-syn+TLK2 CKO, and control mice (n=6 mice for each genotype). Quantification of the total distance and mean speed for 5 minutes in the field. (**C**) Immunostaining images of the TH neurons (red) in the SNc, nuclei were stained with DAPI (blue), α-synuclein were accompany with GFP fluorescent protein (green) (n=4 mice for each genotype). Scale bar, 100 µm.

**Supplementary video**

The video displayed the mobility of GluR1^Lc^ and GluR1^Lc^ / TLK2 CKO mice after 5 days of dox treatment. GluR1^Lc^ mice showed defective mobility; whereas GluR1^Lc^ / TLK2 CKO mice displayed better mobility (n=6 mice for each genotype).

**Supplementary Table legends**

**Table S1. Genetic screens using UAS-RNAi and candidate-based lines in the calcium overload model.**

**Table S2.** **The list of identified genetic modifiers in the calcium overload model.**
