# Supplementary figures and images for: Calcium overload induced mitochondrial and lysosomal dysfunction is regulated by Tousled-like kinase in a-synucleinopathy
Source: Cell Death Dis. 2026 Jan 8;17(1):10. doi: 10.1038/s41419-025-08213-8 (PMC12783599; doi:10.1038/s41419-025-08213-8)

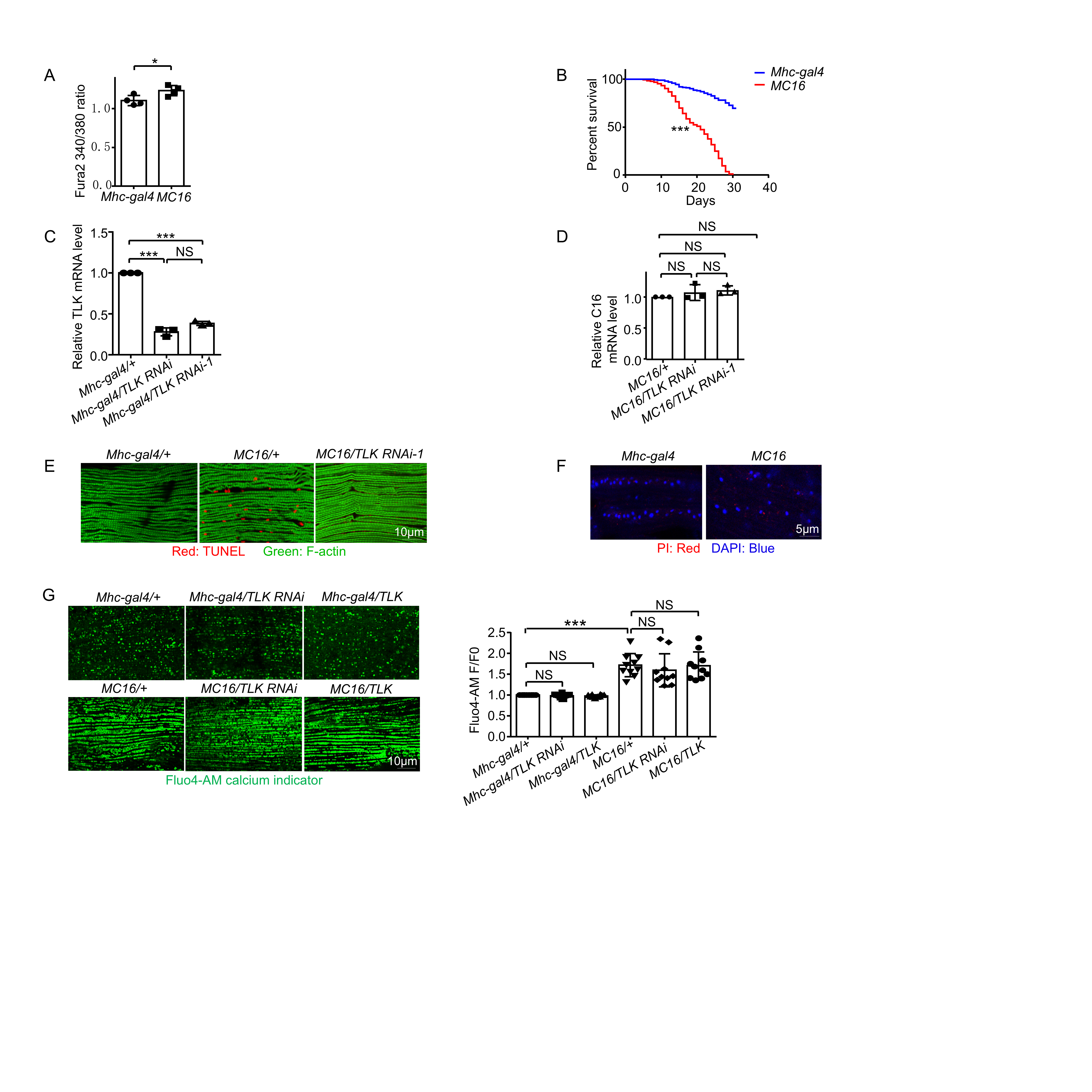

Supplement: Supplementary file 2 — Figure S1 [file 41419_2025_8213_MOESM2_ESM.tif]

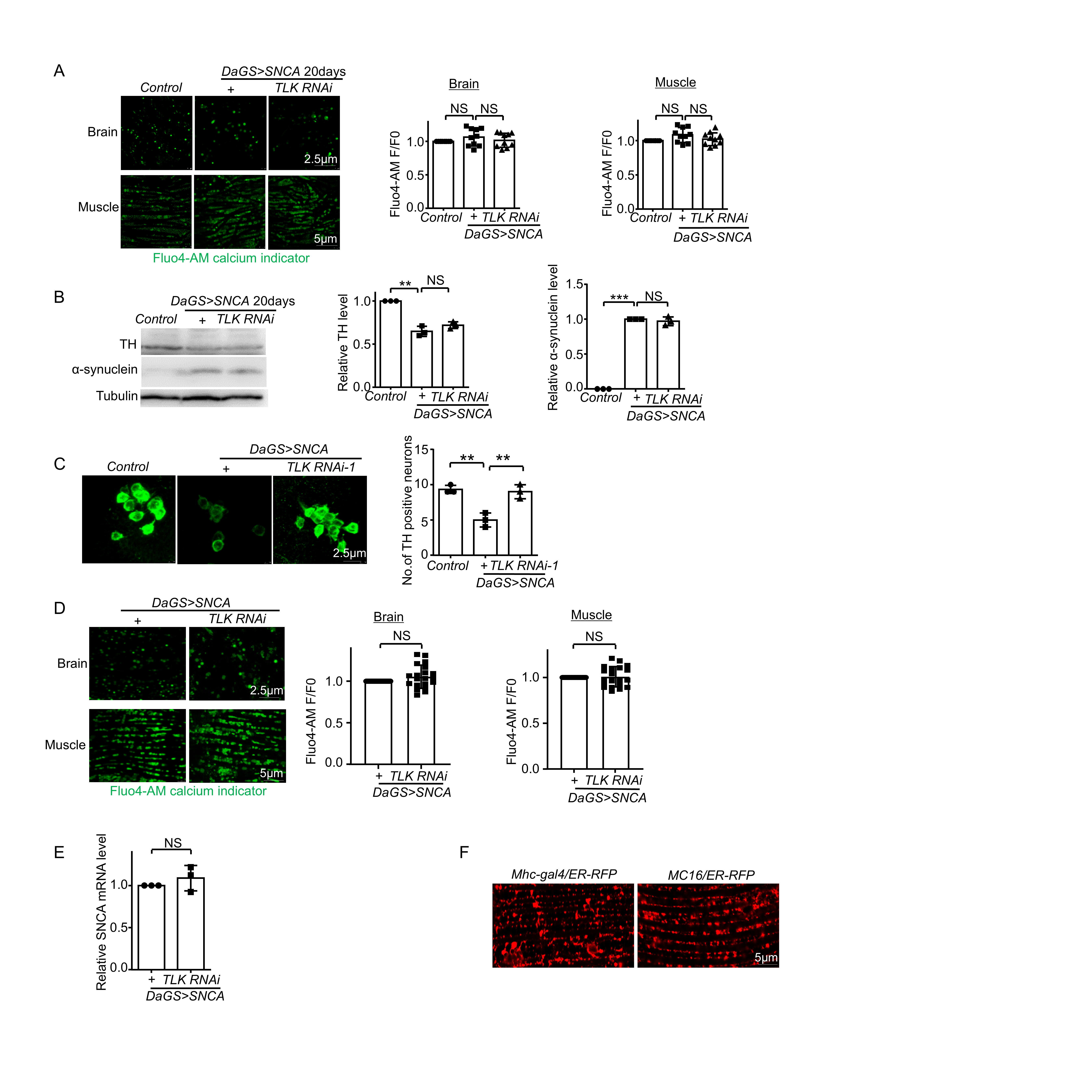

Supplement: Supplementary file 3 — Figure S2 [file 41419_2025_8213_MOESM3_ESM.tif]

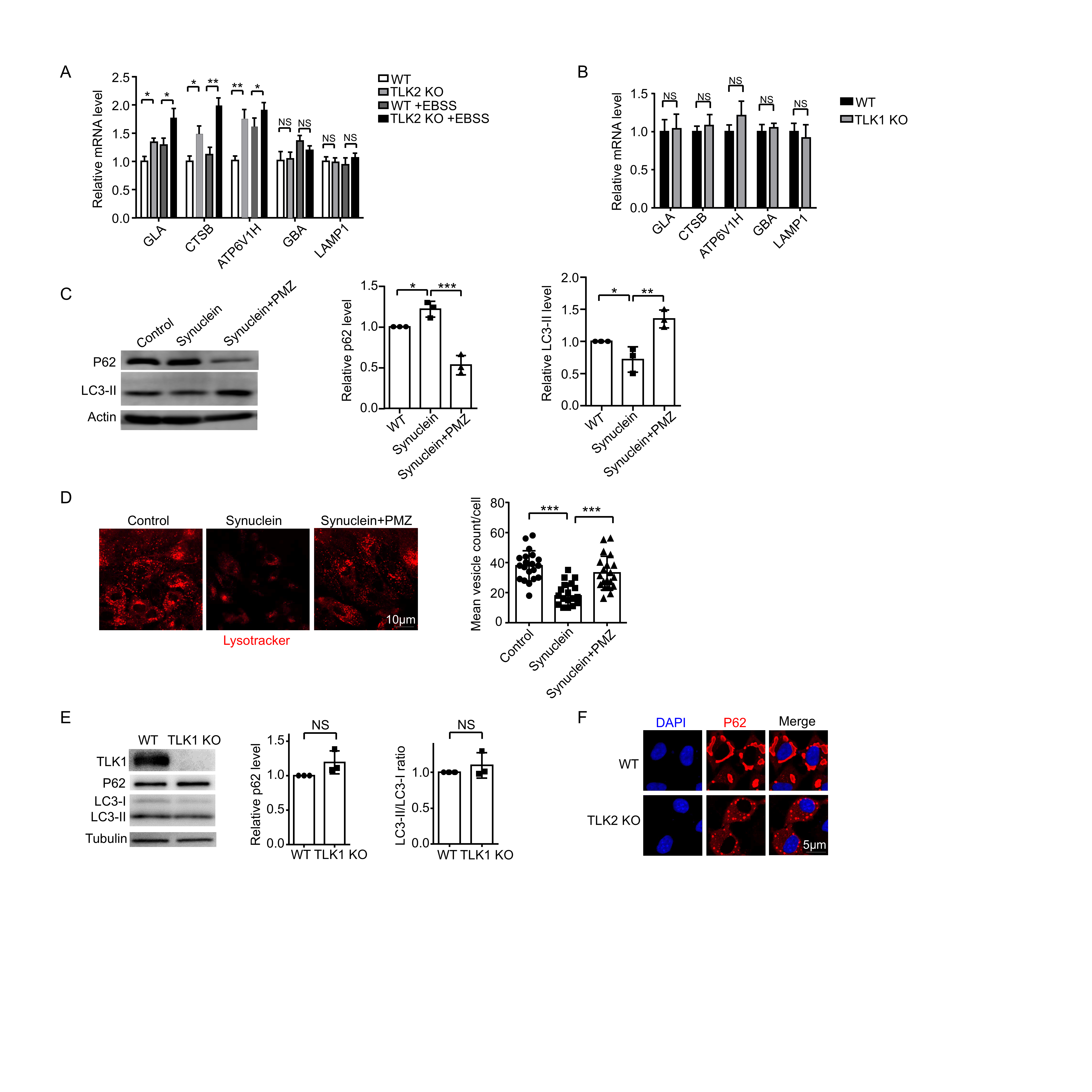

Supplement: Supplementary file 4 — Figure S3 [file 41419_2025_8213_MOESM4_ESM.tif]

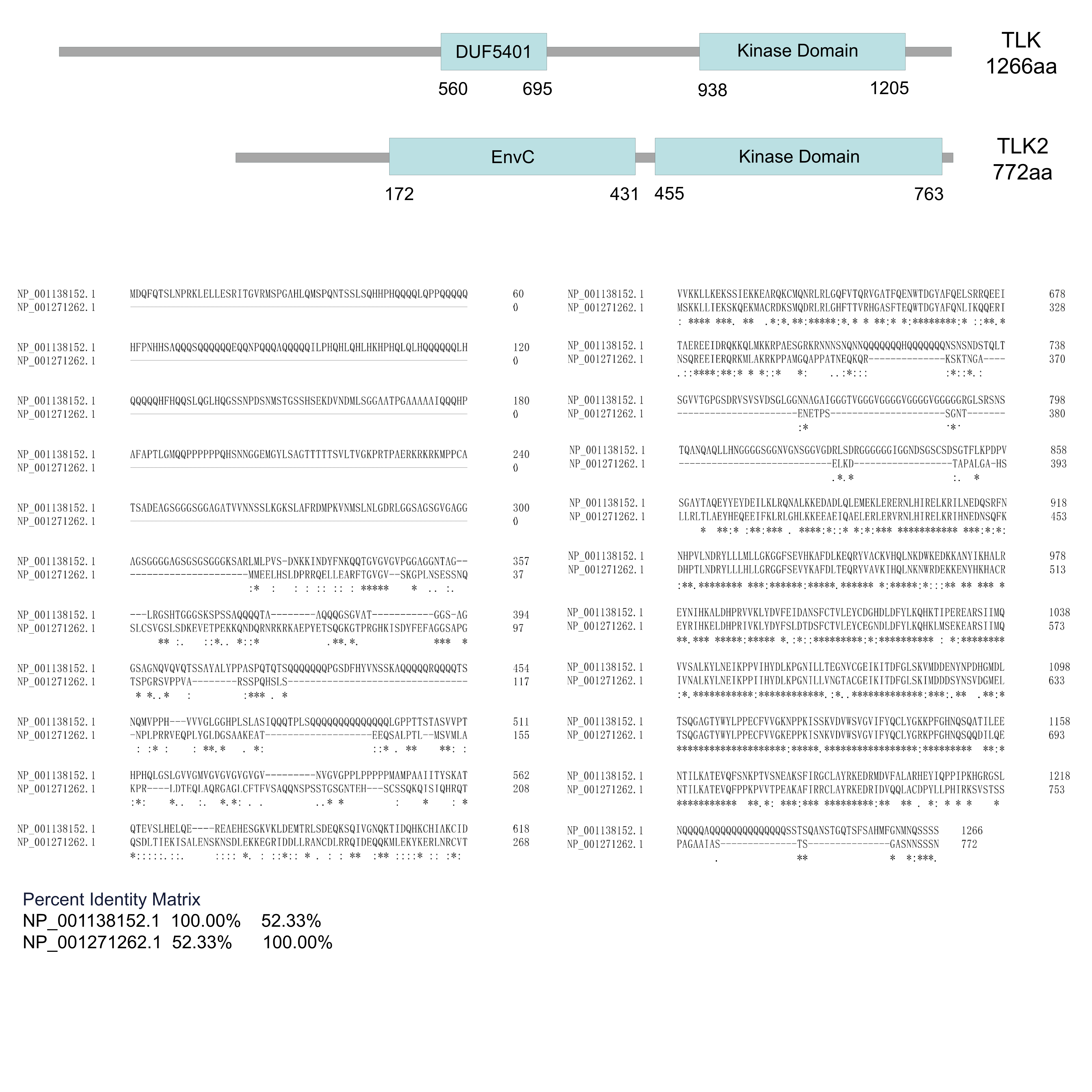

Supplement: Supplementary file 5 — Figure S4 [file 41419_2025_8213_MOESM5_ESM.tif]

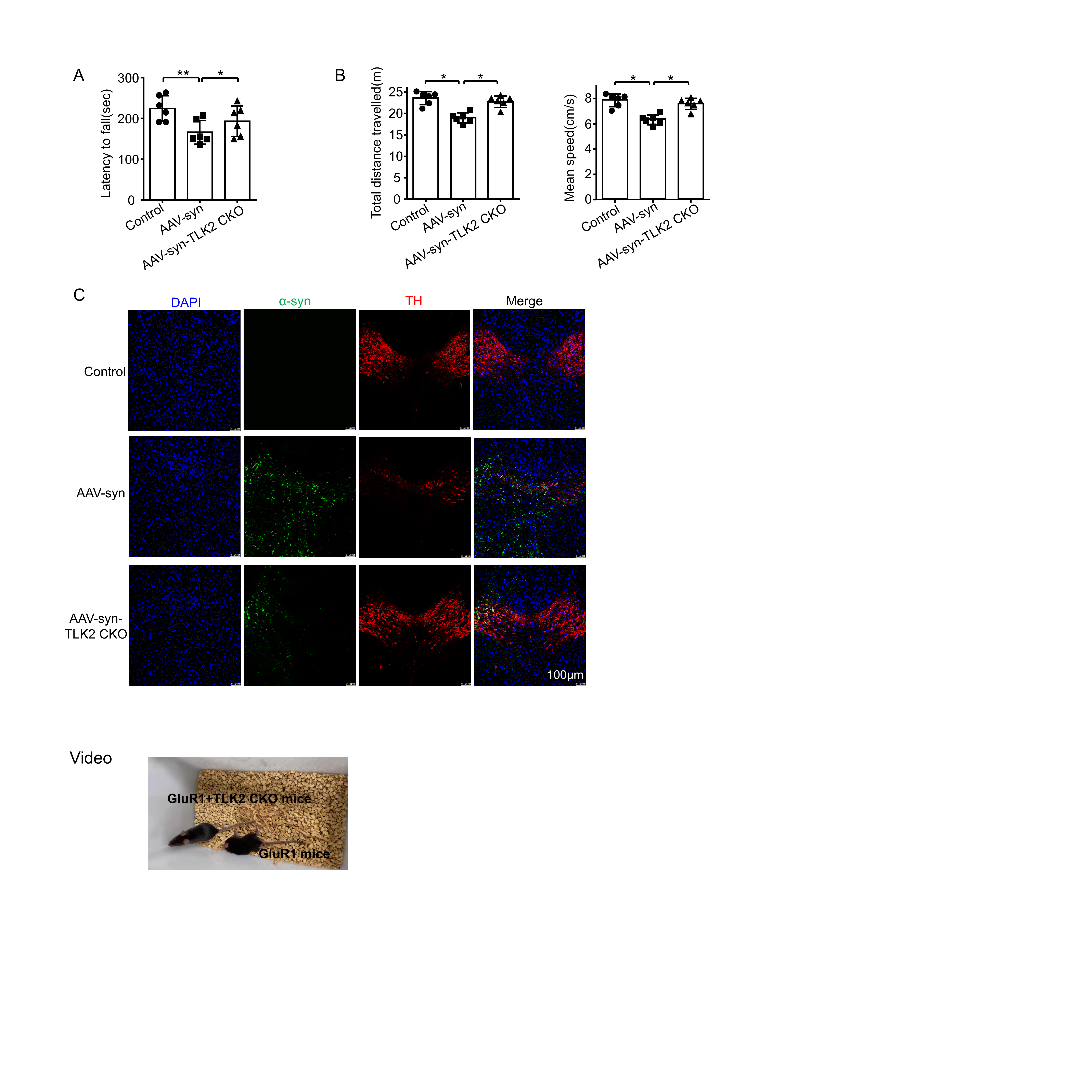

Supplement: Supplementary file 6 — Figure S5 [file 41419_2025_8213_MOESM6_ESM.tif]

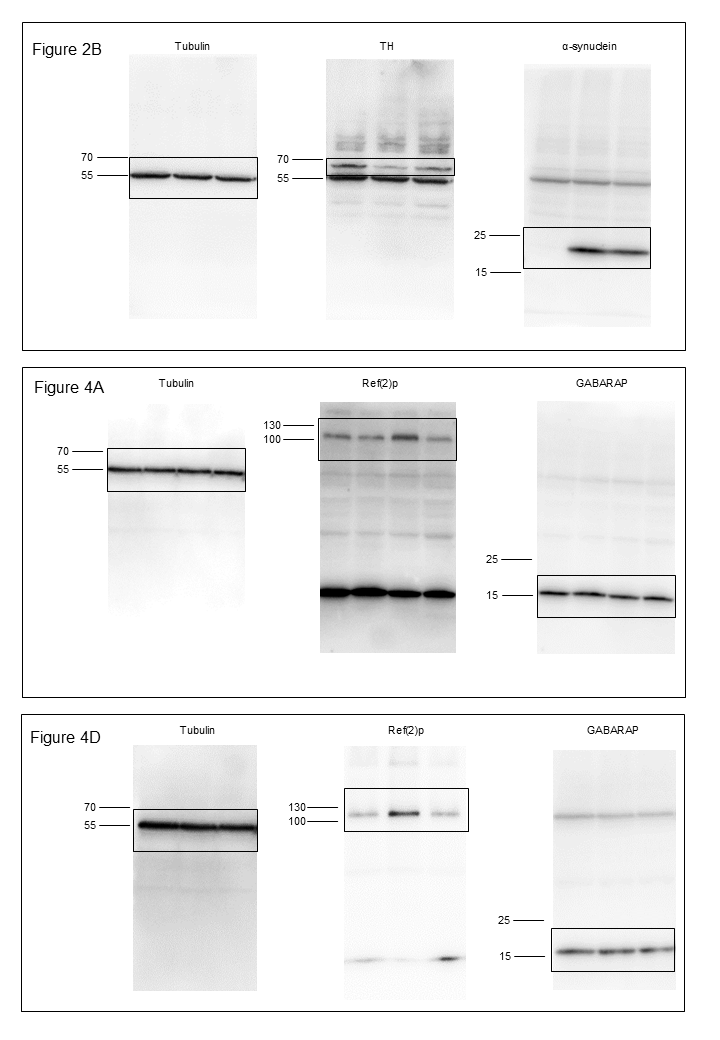

Supplement: Supplementary file 9 — original data [file 41419_2025_8213_MOESM9_ESM.zip › original data/uncropped western blots-1.TIF]

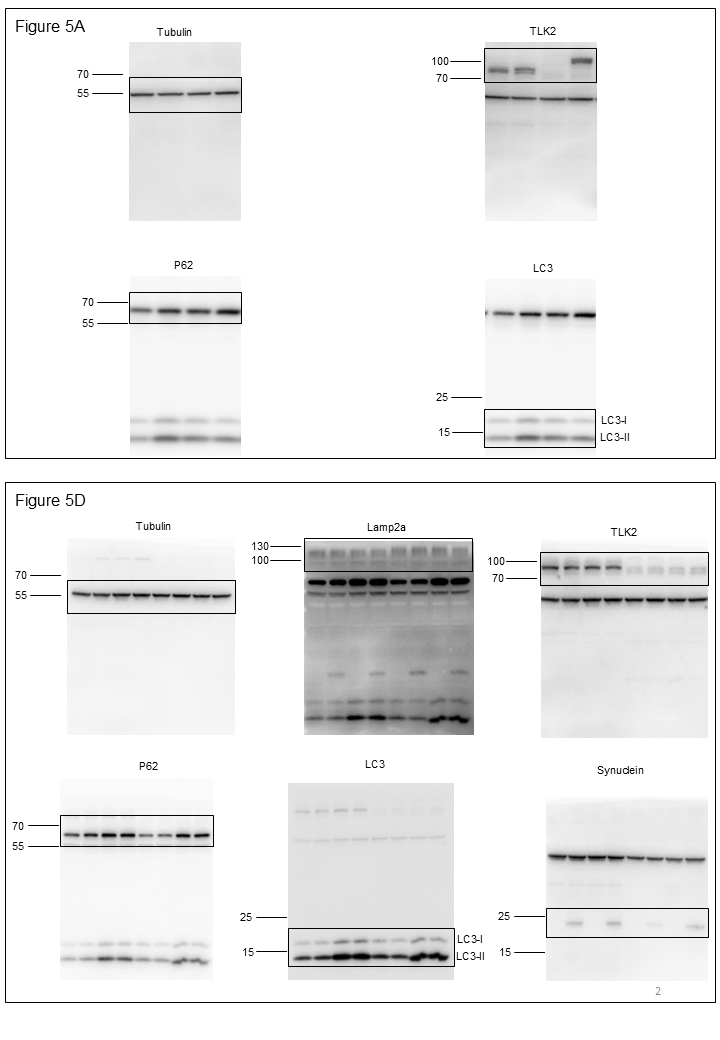

Supplement: Supplementary file 9 — original data [file 41419_2025_8213_MOESM9_ESM.zip › original data/uncropped western blots-2.TIF]

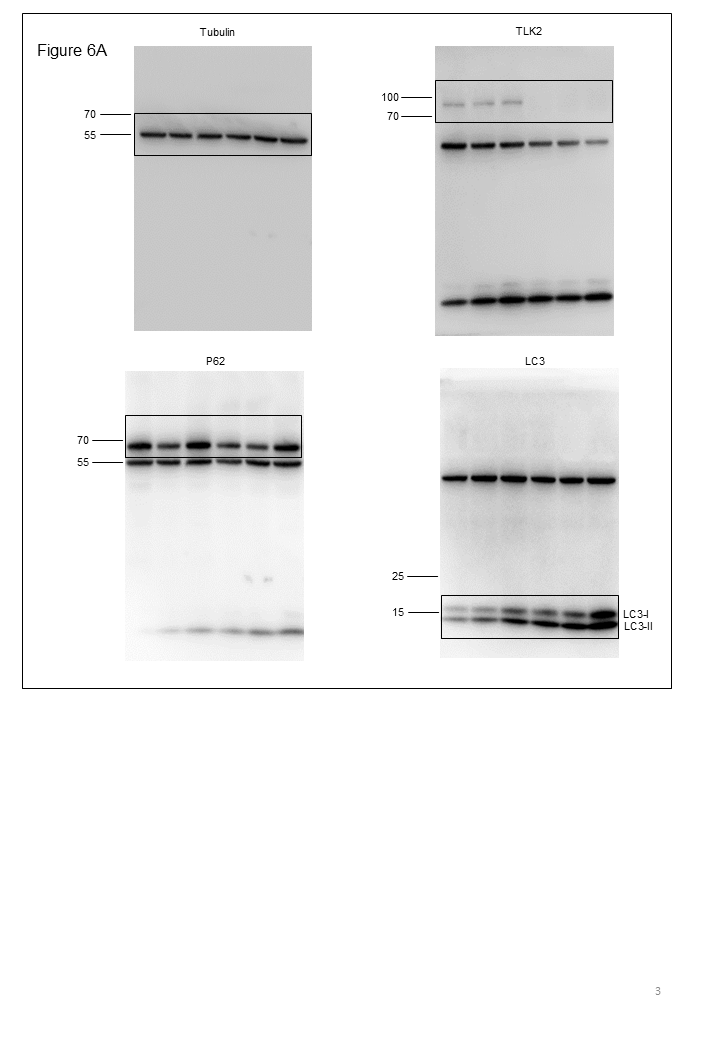

Supplement: Supplementary file 9 — original data [file 41419_2025_8213_MOESM9_ESM.zip › original data/uncropped western blots-3.TIF]

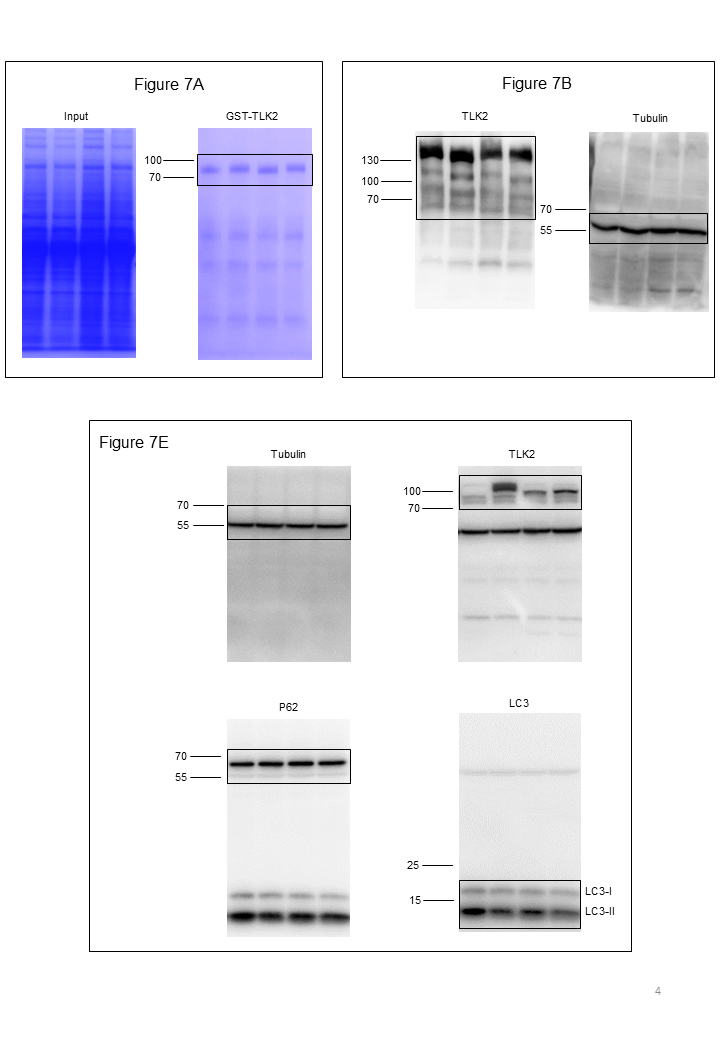

Supplement: Supplementary file 9 — original data [file 41419_2025_8213_MOESM9_ESM.zip › original data/uncropped western blots-4.TIF]

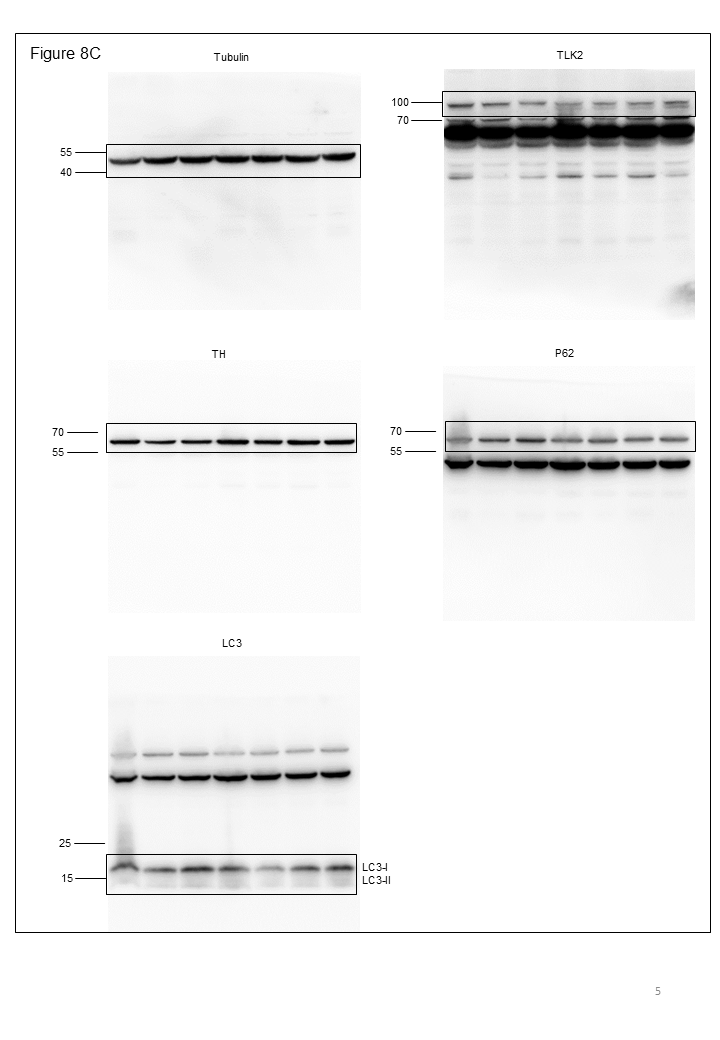

Supplement: Supplementary file 9 — original data [file 41419_2025_8213_MOESM9_ESM.zip › original data/uncropped western blots-5.TIF]

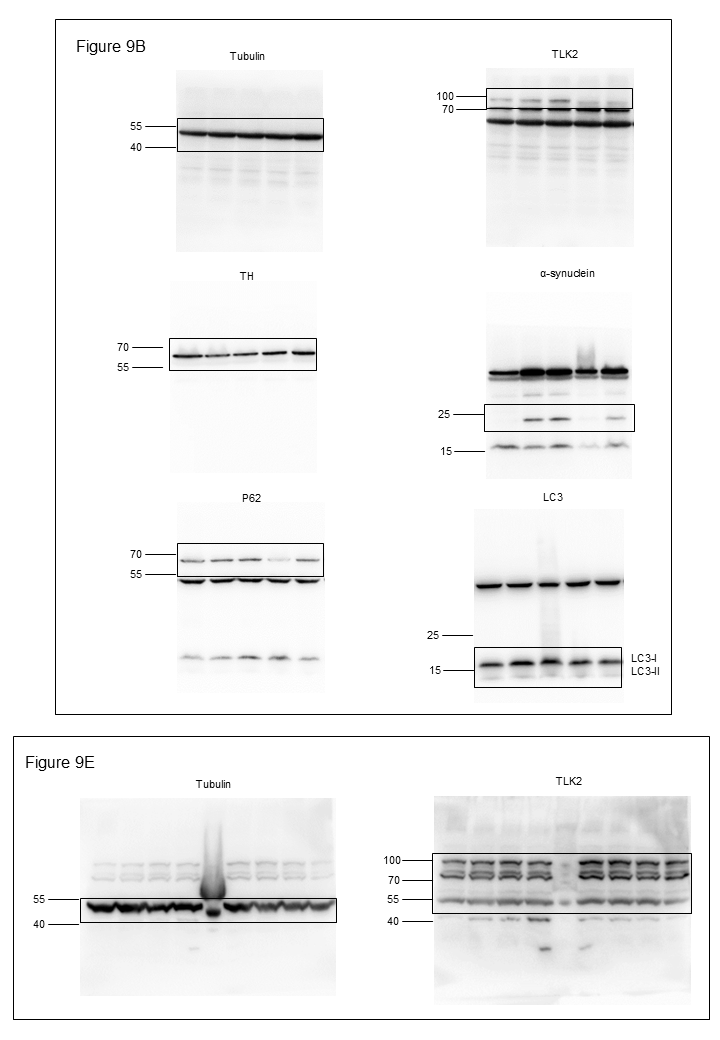

Supplement: Supplementary file 9 — original data [file 41419_2025_8213_MOESM9_ESM.zip › original data/uncropped western blots-6.TIF]

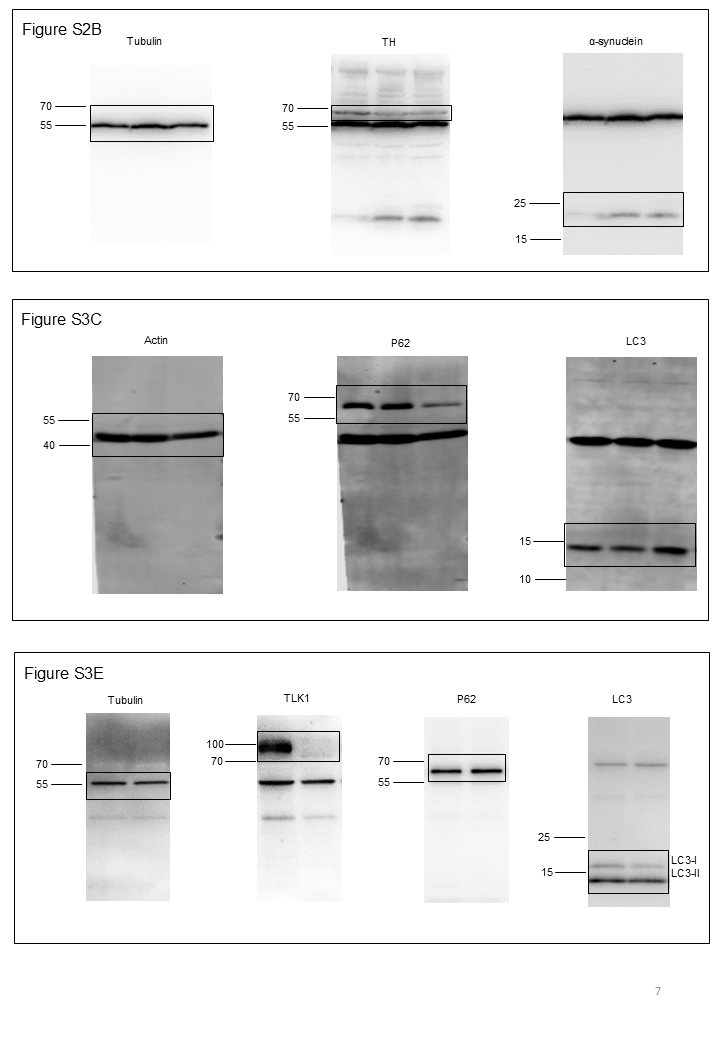

Supplement: Supplementary file 9 — original data [file 41419_2025_8213_MOESM9_ESM.zip › original data/uncropped western blots-7.TIF]
